# Exon skipping for Duchenne muscular dystrophy: a systematic review and meta-analysis

**DOI:** 10.1186/s13023-018-0834-2

**Published:** 2018-06-15

**Authors:** Yuko Shimizu-Motohashi, Terumi Murakami, En Kimura, Hirofumi Komaki, Norio Watanabe

**Affiliations:** 1 Department of Child Neurology, National Center Hospital, National Center of Neurology and Psychiatry, 4-1-1 Ogawahigashi-cho, Kodaira, Tokyo, 187-8551 Japan; 2 Department of Neurology, National Hospital Organization Higashisaitama Hospital, 4147 Kurohama, Hasuda, Saitama, 349-0196 Japan; 3 Extra Early Exploratory Clinical Trial Unit, Translational Medical Center, National Institute of Neuroscience, National Center of Neurology and Psychiatry, 4-1-1 Ogawahigashi-cho, Kodaira, Tokyo, 187-8551 Japan; 4 Department of Health Promotion and Human Behavior, Graduate School of Medicine/School of Public Health, Kyoto University, Yoshida Konoe-cho, Sakyo-ku, Kyoto, 606-8501 Japan

**Keywords:** Eteplirsen, Drisapersen, Randomized controlled trial, Pooled estimates, 6-min walk test, Prospectively planned meta-analysis, Real-world data, Rare disease

## Abstract

**Background:**

Exon skipping has been considered a promising therapeutic approach for Duchenne muscular dystrophy (DMD). Eteplirsen received conditional approval in the United States in 2016. To date, no systematic reviews or meta-analyses of randomized controlled trials (RCTs) of exon skipping drugs have been published to determine the pooled estimates for the effect of exon skipping in treating DMD.

**Methods:**

A systematic review and meta-analysis of double-blind RCTs comparing exon-skipping drugs with placebo in DMD was performed. Trials were identified by searching published and unpublished studies from electronically available databases and clinical trial registries through October 2017. The primary outcomes were changes in the 6-min walk test (6MWT) distance, North Star Ambulatory Assessment (NSAA) scores, and adverse events. Random-effects meta-analysis and assessment of risk of bias were performed. This systematic review was registered at PROSPERO (CRD42016037504).

**Results:**

Five studies involving 322 participants were included, investigating eteplirsen in one and drisapersen in four studies. There were no changes in 6MWT distance (mean difference [MD] − 9.16, 95% confidence interval [CI] − 21.94 to 3.62) or NSAA scores (MD 1.20, 95% CI − 2.35 to 4.75) after 24 weeks of treatment in the exon-skipping group compared with placebo. Subgroup analysis for a 6 mg/kg weekly injection of drisapersen showed significant changes in the 6MWT, favoring drisapersen after 24 weeks (MD − 20.24; 95% CI − 39.59 to − 0.89). However, drisapersen resulted in a significant increase in injection site reactions (risk ratio [RR] 3.67, 95% CI 1.96 to 6.89, *p* < 0.0001) and renal toxicity (RR 1.81, 95% CI 1.11 to 2.94, *p* = 0.02). Risk of bias was high in two of the five studies, including the eteplirsen and one drisapersen study.

**Conclusions:**

Current available data do not show evidence that exon-skipping drugs are effective in DMD. Despite potential effectiveness when used at a specific dose, significant side effects were reported with drisapersen. The small number of RCTs with relatively small numbers of participants indicate the difficulty in conducting sufficiently powered studies of DMD. Prospectively planned meta-analysis and utilization of the real-world data may provide a more precise estimate of the effect of exon skipping in this disease.

**Electronic supplementary material:**

The online version of this article (10.1186/s13023-018-0834-2) contains supplementary material, which is available to authorized users.

## Background

Duchenne muscular dystrophy (DMD) is a rare, childhood-onset, progressive muscular disorder caused by a mutation in the *DMD* located on Xp21 [1]. It is estimated to affect one in 3500 to 6000 live male births [2–4]. Individuals with DMD become wheel-chair bound before or during their teens and patients eventually develop respiratory and cardiac dysfunction [5, 6]. Currently, there is no curative therapy for DMD.

Among several therapeutic approaches being investigated for this disorder is the exon skipping drug eteplirsen, for which the U.S. Food and Drug Administration (FDA) announced accelerated approval in September 2016 [7]. Exon skipping is induced by antisense oligonucleotides (AOs). This approach is based on the rationale that converting the translational reading frame for the mutated dystrophin protein from out-of-frame to in-frame produces a shorter but functional dystrophin in place of the nonfunctioning dystrophin seen in DMD [8].

Despite a number of studies demonstrating significant success in treating DMD in animal models [9, 10], several clinical trials of exon skipping have failed to demonstrate clear efficacy [11]. Given the relatively small number of patients with DMD, it is often difficult to conduct a rigorous study with sufficient power. Several approaches have been proposed to overcome the methodologic challenges in studies of rare diseases, one solution being to combine the results of different trials and perform a meta-analysis of the data [12]. Herein, we describe the results of a systematic review and meta-analysis of randomized controlled trials (RCTs) of exon-skipping drugs in DMD, assessing their efficacy and limitations.

## Methods

A systematic review of available literature was conducted in compliance with the PRISMA statement (Additional file 1: Appendix 1) [13]. The objective was to determine whether exon skipping therapies can positively change the clinical course in patients with DMD. Standardized review and extraction protocols were developed a priori (Additional file 1: Appendix 2). The study was registered with PROSPERO (CRD42016037504).

### Selection criteria

Reports describing double-blind RCTs and the first phase of controlled crossover trials were evaluated for the review. Participants were included in the reviewed studies if they were confirmed to have out-of-frame *DMD* mutations deemed by the authors as correctable by exon skipping.

The exon-skipping agents searched for are described in our study protocol (Additional file 1: Appendix 2). All drug administration regimens were included for review. Effects were compared between the exon-skipping and placebo groups, and concomitant usage of glucocorticoids (GCs) was accepted only if both the treated and control groups were given GCs that had been started prior to initial administration of the exon-skipping drug.

The study outcomes assessed in the review were determined by personal communication with parents whose child had DMD as well as with patients themselves, based on discussions among the authors. The primary outcomes assessed at week 24 of treatment were a change from baseline in the distance covered during the 6-min walk test (6MWT) and in the North Star Ambulatory Assessment (NSAA) score, as well as adverse events (AEs) occurring during the study period. Secondary outcomes were defined as the change in the 6MWT distance from baseline to week 48 of treatment, change in time taken for timed tests from baseline to week 24, change in quality of life (QOL) score from baseline to week 48, and survival.

### Search strategy

Using pre-specified search terms (Appendices 3–6), we electronically searched the following databases through May 22, 2016: Cochrane Central Register of Controlled Trials (CENTRAL), MEDLINE with Ovid, EMBASE at embase.com, and ICHUSHI-web (Japana Centra Revuo Medicina). The search results were updated in October and November 2017 (CENTRAL on October 23, 2017; MEDLINE on October 26, 2017; EMBASE on November 29, 2017; ICHUSHI-web on October 24, 2017). Clinical trials were also identified by a hand-search of the Primary Registries in the WHO Registry Network and in registries approved by the International Committee of Medical Journal Editors on September 8, 2016, using prespecified search terms (Additional file 1: Appendix 7); this search was updated on October 19, 2017. There were no restrictions on publication language or status, and both peer reviewed and non-peer reviewed publications were included. We also contacted groups planning to conduct a relevant RCT and screened the bibliography of all retrieved manuscripts to check for studies not identified by the original search. For large clinical trials sponsored by pharmaceutical companies known to us but not reported, information was obtained either from the pharmaceutical company website or by direct contact.

### Data extraction

Two authors (YSM and TM) independently reviewed the titles, abstracts, and the full text of all the retrieved articles to determine their eligibility. The authors were not blinded prior to assessment.

YSM and TM independently assessed the studies’ relevance and extracted data onto a previously agreed upon data extraction form [14]. YSM and TM also independently assessed the risk of bias pertaining to sequence generation, allocation concealment, blinding of participants, blinding of outcome assessors, incomplete outcome data, selective outcome reporting, and other sources of bias as previously described [15]. Risk of bias was assessed at the study and outcome levels according to previously described methodology [15]. Any disagreement was resolved either by a discussion between YSM and TM or, if necessary, with EK and NW.

### Data analysis

We analyzed dichotomous data as risk ratios (RRs) and continuous data as mean differences (MD) or as standardized mean difference. We reported these measures of effect with their 95% confidence intervals (CI).

We undertook meta-analyses with the Cochrane statistical software RevMan [16], and used random-effects meta-analyses for comparison. Where a single trial included multiple trial arms, we combined all the trial arms statistically into one arm and compared those results with that of the placebo arm. If standard deviations (SDs) were not reported for continuous data in the original publication, SDs reported by other studies were substituted [17].

For missing or unreported data, the original investigators were contacted and requested to provide the relevant data. If data were missing for a continuous variable, suitable methods such as mixed-effect models for repeated measures were used to impute the missing data.

Heterogeneity in intervention effects among the trials was statistically tested using the standard Chi^2^ statistic (*p* value) and the Higgins I^2^ statistic expressed as a percentage; *p* values of less than 0.1 were taken as evidence of heterogeneity. Interpretations of statistical heterogeneity were made based on established recommendations [15].

Any substantial but unexplained heterogeneity that was identified was reported, and possible causes were explored using prespecified subgroup analysis (Additional file 1: Appendix 2). Sensitivity analyses to assess the effects of including studies with a high risk of bias were performed by repeating the meta-analysis after excluding any or all studies with that high risk. The quality of a body of evidence was assessed based on the Grading of Recommendations, Assessment, Development and Evaluation (GRADE) considerations of study limitations, consistency of effect, imprecision, indirectness, and publication bias. The summary of findings table was created using GRADEpro GDT software [18].

## Results

**Fig. 1:**
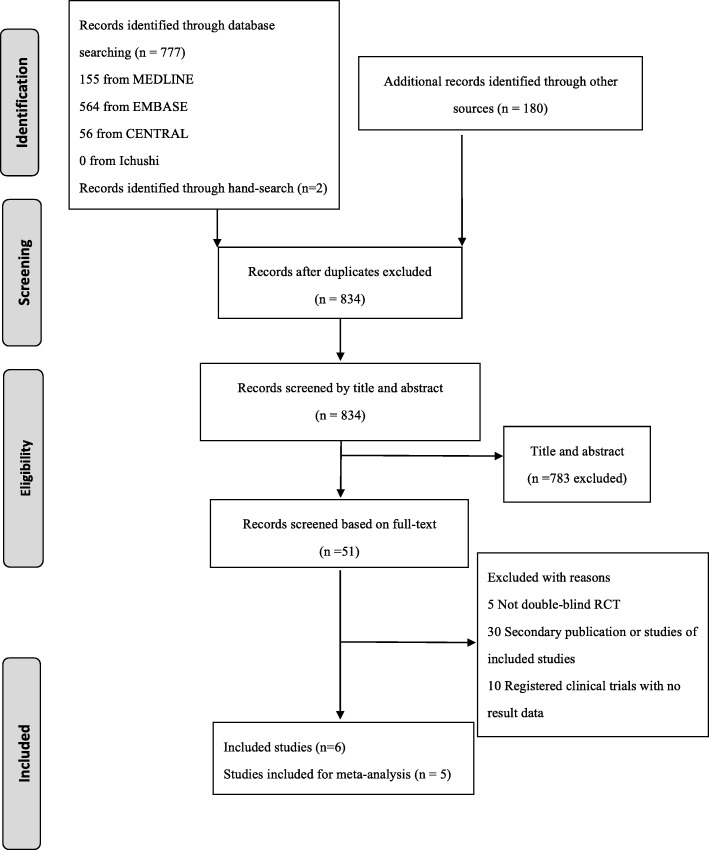
Flow diagram depicting the process of study selection

The electronic search of all listed databases and the hand-search of clinical trial registries and publications identified 957 records (Fig. 1), with 834 records remaining after exclusion of duplicates. These were screened based on title and abstract for database records and by title and registration information for registered clinical trials, yielding 51 publications. Of these, five full-text articles were not RCTs, 30 were secondary publications, and 10 were registered clinical trials but had no reported results (Additional file 1: Appendix Table 1). Therefore, a total of six studies were eligible for the review. However, one study could not be included in the meta-analysis as no data relevant to the outcomes being analyzed in this review were reported [19]. Finally, five studies involving 322 participants were included for quantitative synthesis (meta-analysis) (Table 1) [20–24].

**Table 1:** Characteristics of included studies

Study ID (Study Period) Funding source	Number radomized, countries	Mean age of participants y (SD, range)	Mean FVC at baseline (SD)	Mean 6MWT at baseline meters (SD)	Interventions (n)	Co-intervention with glucocorticoids	Treatment duration	Observation period	Outcome assessed
**Flanigan 2014** [24] **(2010–2011)** GlaxoSmithKline	20 France USA	12.7 (1.4, 9–16)	NA	NA	Drisapersen, single SC injection 1. 3 mg/kg/dose (6) 2. 6 mg/kg/dose (6) 3. 9 mg/kg/dose (3) 4. Placebo (5)	By trial arm, n (%) 1. 3 mg/kg/dose: 3 (50) 2. 6 mg/kg/dose: 5 (83) 3. 9 mg/kg/dose: 2 (67) 4. Placebo: 3 (60)	Single dose	5 mo	AE^*^ PK Safety and tolerability
**Voit 2014** [20] **(2010–2012)** GlaxoSmithKline, Prosensa Terapeutics BV	53 Belgium France Germany Netherlands Spain Turkey Australia Israel United Kingdom	7.3 (1.5, 5–11)	FVC litres (SD) *n* = 51 1.38 (0.50)	*n* = 53 408.72 (61.61)	Drisapersen 6 mg/kg/time SC Twice/wk. for 3 w followed by below schedule: 1. Continuous (once/wk) (18) 2. Intermittent (twice/wk. at 1,3,5 wk. and once/w at 2,4,6 wk. No drug during 7–10 wk., then the 10-wk cycle was repeated. (17) 3. Placebo (mannitol) (18)	By trial arm, n (%) 1. Continuous: continuous GC 12 (67), intermittent GC 6 (33) 2. Intermittent: continuous GC 9 (53), intermittent GC 8 (47) 3. Placebo: continuous GC 11 (61), intermittent GC 7 (39) Length of GC use prior to the drug, mean mo (SD) 1. Continuous: 26.0 (21.2) 2. Intermittent: 32.6 (17.0) 3. Placebo: 24.2 (14.0)	48 wk	4wk after the last dose	**6MWT at 25 wk** ^*^ 6MWT at 49 wk.^**^ NSAA at 25 wk.^*^ NSAA at 49 wk. AE^*^ Timed test^**^ PedsQL at 25 wk., 49 wk.^**^ Safety and tolerability Myometry Dystrophin level in muscle Production of exon skipped mRNA in muscle biopsy Serum CK Respiratory function Cardiac function Frequency of falls during 6MWT Time to loss of ambulation PK
**NCT01254019** [22] **(2010–2013)** GlaxoSmithKline	186 Argentina Belgium Brazil Canada Chile Czech Republic Denmark France Germany Italy Japan Korea Netherlands Norway Poland Russian Federation Spain Taiwan Turkey	8.2 (2.4)	FVC-% of predicted *n* = 183 87.25 (28.61)	*n* = 186 340.92 (94.49)	Drisapersen, once/wk., SC 1. 6 mg/kg/dose (125) 2. Placebo (61)	NA	48 wk	20wk after the last dose	**6MWT at 48wk** ^**^ 6MWT at 24wk^*^ Timed test^**^ AE^*^ PedsQL at 48 wk.^**^ NSAA at 48wk Myometry Frequency of falls during 6MWT Time to loss of ambulation Serum CK Respiratory function Cardiac function Production of exon skipped mRNA in muscle biopsy PK CGI-I scale HUI score
**Mendell 2013** [23] **(2011–2012)** Sarepta Therapeutics, Muscular Dystrophy Association, Parent Project Muscular Dystrophy, NCRR/NIH, NIH Roadmap for Medical Research	12 USA	8.8 (1.3, 7–10)	NA	*n* = 12 381.93 (51.91)	Eteplirsen, once/wk., IV 1.30 mg/kg/dose (4) 2.50 mg/kg/dose (4) 3.Placebo(PBS) (4)	Usage, n (%) 18-25 mg/d deflazacort 8 (67), 20 mg/d prednisone 1 (8), 25 mg/d prednisone 2 (17), prednisone weekend only 1 (8)	24 wk	Followed by open-label extension study	**%Dystrophin positive fibers** 6MWT at 24wk^*^ 6MWT at 12 wk. AE^*^ Timed test^**^ QOL^**^ NSAA at 12 wk. NSAA at 24 wk.^*^ Quantitative muscle testing
**NCT01462292** [21] **(2011–2013)** GlaxoSmithKline	51 USA	7.8 (2.2)	FVC-% of predicted *n* = 48 98.6 (14.14)	n = 51 408.87 (58.66)	Drisapersen, once/wk., SC 1.3 mg/kg/dose (17) 2.6 mg/kg/dose (18) 3.Placebo (16)	NA	24 wk	24 wk. after the last dose	**6MWT at 24 wk** ^*^ Timed test^**^ NSAA at 24 wk.^*^ AE^*^ Frequency of falls during 6MWT Serum CK Respiratory function Myometry CGI-I Dystrophin expression in muscle PK

mo = months, wk. = week, SC = subcutaneous, 6MWT = 6 min walk test, NSAA = North Star Ambulatory Assessment, IV = intravenous, PBS = phosphate buffered saline, GC = glucocorticoids, NA = not available, AE = adverse events, PK = pharmacokinetics, CK = creatine kinase, CGI-I=Clinical Global Impression of Improvement, HUI=Health Utilities Index

Bold indicates primary outcome of each study. * indicates primary outcome for the current meta-analysis. ** indicates secondary outcome for the current meta-analysis

**Fig. 2:**
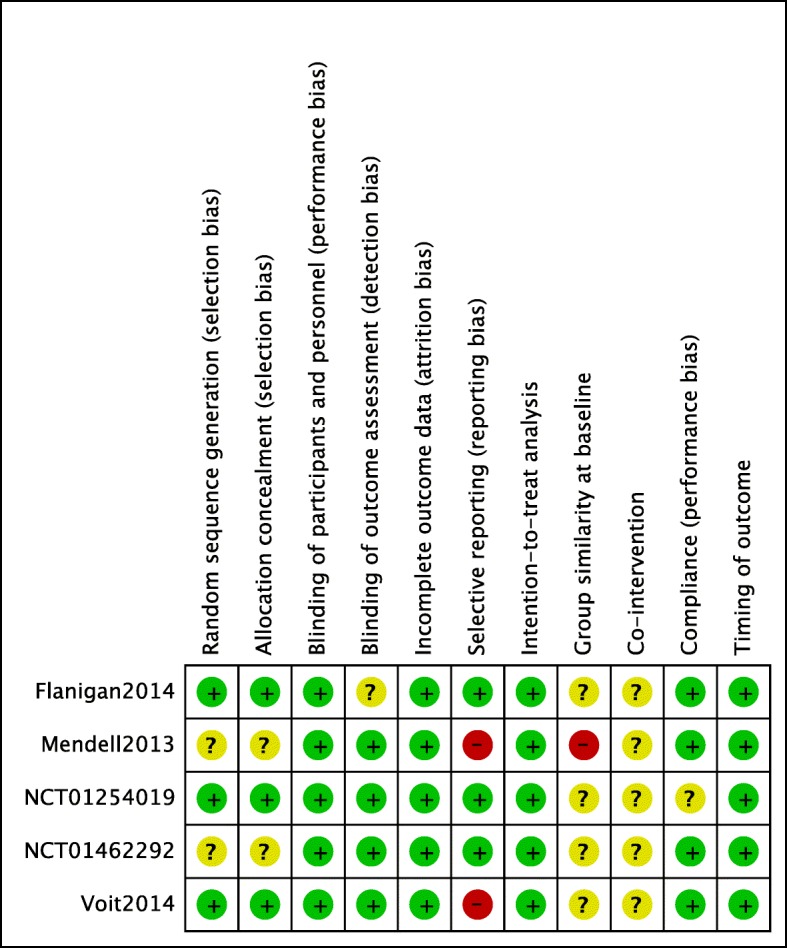
Risk of bias summary. A review of the authors’ judgment on each category of risk of bias for each of the five studies included is shown

The risk of bias assessed at the study level is shown in Fig. 2. All five studies were double-blind RCTs, but only Flanigan 2014 [24], Voit 2014 [20], and NCT01254019 [22] provided detailed information on randomization and allocation concealments. Information on blinding of participants, personnel, and outcome assessment was available for all trials, except for that of Flanigan 2014 [24]. Data on the 6MWT were missing in the Voit 2014 [20] and NCT01254019 [22], but these omissions accounted for less than 20% of the total data. The other three studies (Flanigan 2014 [24], NCT01462292 [21], Mendell 2013 [23]) had no missing data for the 6MWT. Selective reporting bias was considered to be high in two of the RCTs; no information on pre-specified primary outcomes was available in Voit 2014 [20], and the study by Mendell 2013 [23] did not provide data on changes in QOL despite stating that it had been assessed. There was insufficient information to judge group dissimilarity bias at baseline in four studies (Flanigan 2014 [24], Voit 2014 [20], NCT01254019 [22], NCT01462292 [21]). We judged the RCT by Mendell 2013 [23] to be at high risk for group dissimilarity bias because a pair of identical twins was allocated to the 30 mg/kg exon skipping group, and both patients had rapid disease progression. Information on co-interventions, including use of GCs or physical therapy, was insufficient in all five studies. Information on compliance was considered to be insufficient to assess performance bias in NCT01254019 [22], taking into account the fact that this trial involved a large number of participants from many countries. We found that there was no bias in terms of incomplete outcome data or timing of outcome in any of the studies. An intention-to-treat analysis had been performed in all studies.

The risk of bias in 6MWT measurements was assessed in all studies except that of Flanigan 2014 [24], which had no data for the 6MWT. Performance, detection, and attrition bias were considered low risk in the other four studies.

**Fig. 3:**
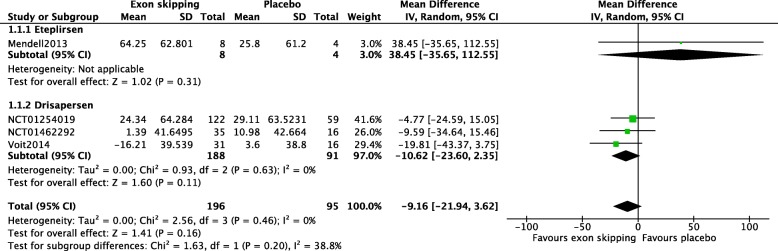
Change in the 6MWT at week 24. Change from baseline (meters) in distance covered by the 6MWT measured after 24 weeks of treatment. In the figure, the mean difference of gain in distance is shown in negative numbers and loss in distance is shown in positive numbers, i.e., 64.25 indicates − 64.25 for actual measurement

Four studies involving a total of 291 participants had data on the 6MWT after 24 weeks of intervention (Fig. 3). No significant difference in the change in distance covered in the 6MWT from baseline to week 24 of treatment was found between the exon-skipping and placebo groups (MD − 9.16, 95% CI − 21.94 to 3.62). Moderate heterogeneity was observed between the eteplirsen and drisapersen studies (I^2^ = 38.8%, Chi^2^ = 1.63, *p* = 0.2). Notably, when changes in the 6MWT in placebo groups across the different studies were assessed, placebo groups in all four studies showed decline in the 6MWT from baseline at week 24 of treatment (Mendell 2013: mean change − 25.8 m, SD 61.2; NCT01254019: mean change − 29.11 m, SD 63.523; NCT01462292: mean change − 10.98 m, SD 42.664; Voit 2014: mean change − 3.6 m, SD 38.8).

**Fig. 4:**
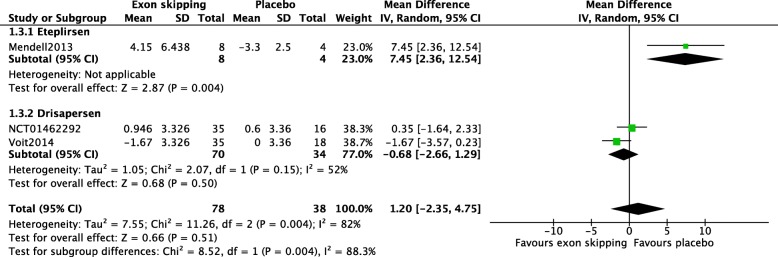
Change in NSAA score at week 24. Change in NSAA score from baseline to after 24 weeks of treatment. In general, a higher score indicates better motor function. For the mean difference, gain of score is shown in negative numbers and loss of score in positive numbers in the figure, i.e., 4.15 indicates a negative score of − 4.15 in the actual measurement. As the trial by Voit2014 had only provided the adjusted mean difference versus placebo, SDs for each intervention group could not be calculated and were substituted with SDs reported in the NCT01462292 trial. There was no significant change in the NSAA scores between the treated and placebo groups. Subgroup analysis revealed a significant increase in the scores in the placebo groups compared with those in the eteplirsen group

Data on change in NSAA score from baseline to after 24 weeks of intervention was available in three RCTs (Fig. 4) with a total of 116 participants. There was no significant difference in change in NSAA score from baseline to at week 24 (MD 1.20; 95%CI − 2.35 to 4.75), and considerable heterogeneity was observed between the eteplirsen and drisapersen studies (I^2^ = 88.3%, Chi^2^ = 8.52, *p* = 0.004).

**Fig. 5:**
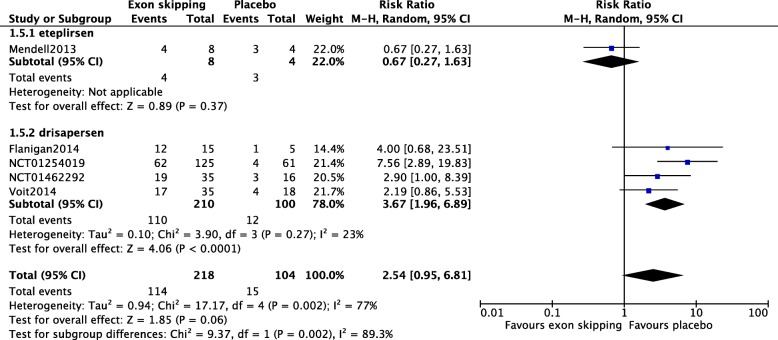
Title: Adverse events, injection site reactions. There was no significant difference in the injection site reaction between the treated and placebo groups. Subgroup analysis revealed a significant increase in the drisapersen group, but not in the eteplirsen group, compared with that in the placebo groups

**Fig. 6:**
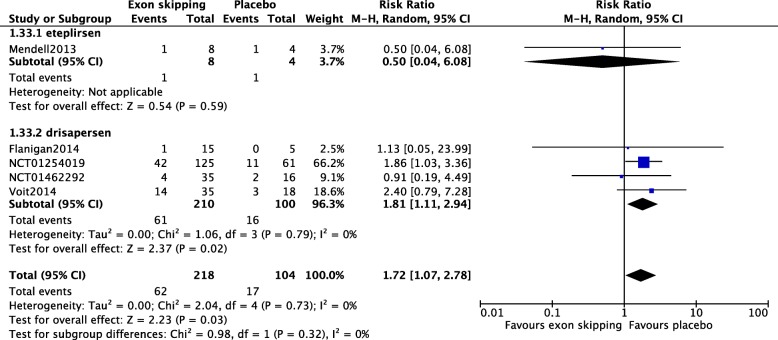
Adverse events, renal toxicity. Subgroup analysis showed a significant increase in the renal toxicity in the drisapersen group, but not in the eteplirsen group, compared with that in the placebo groups

Data on AEs were available in all five studies. Overall, there was no significant difference in injection site reaction between the exon skipping and the placebo groups (Risk Ratio (RR) 2.54; 95%CI 0.95 to 6.81) (Fig. 5). However, when subgroup analysis was performed, studies of drisapersen revealed a significantly higher number of participants with injection site reaction with the drug compared with placebo (RR 3.67; 95%CI 1.96 to 6.89) (Fig. 5). Substantial heterogeneity was observed in the numbers of participants with injection site reactions among all five studies (I^2^ = 77%, Chi^2^ = 17.17, *p* = 0.002), as well as between those of eteplirsen and the drisapersen (I^2^ = 89.3%, Chi^2^ = 9.37, *p* = 0.002). Compared with placebo, administration of an exon skipping drug was associated with a higher incidence of renal toxicity (RR 1.72; 95%CI 1.07 to 2.78) (Fig. 6), but subgroup analysis demonstrated that this was only true in the drisapersen studies (RR 1.81; 95%CI 1.11 to 2.94) but not in the eteplirsen trial (Fig. 6). Data on all other AEs are shown in appendices 8 to15.

The data provided on all secondary outcomes tested showed no significant differences between the exon-skipping and placebo groups (Additional file 1: Appendix 16–22). None of the RCTs reported survival data.

The number of participants who withdrew from a study did not differ significantly between the exon-skipping and placebo groups (Additional file 1: Appendix 23).

Subgroup analysis to compare eteplirsen versus placebo (Additional file 1: Appendix 24) or drisapersen versus placebo (Additional file 1: Appendix 25) showed a nonsignificant difference in the 6MWT results. In contrast, subgroup analysis to compare the effect of either eteplirsen or drisapersen on the NSAA score demonstrated significantly better scores in the placebo group compared with the eteplirsen group (Mendell 2013 [23]; Additional file 1: Appendix 26, MD 7.45 95%CI 2.36 to 12.54), whereas there were no significant differences between the drisapersen and the placebo groups (Additional file 1: Appendix 27).

The dose of drisapersen administered varied among the studies, with a once-weekly 6 mg/kg injection being the most common. Subgroup analysis for this drisapersen regimen showed significant improvement in the 6MWT at 24 weeks favoring the drisapersen group (MD − 20.24; 95%CI − 39.59 to − 0.89) (Additional file 1: Appendix 28), with a longer 6MWT distance in the drisapersen group. However, there were significantly more number of patients with injection site reactions (RR 3.71, 95%CI 1.93 to 7.15) and renal toxicity (RR 1.83, 95%CI 1.10 to 3.03) in this group (Additional file 1 : Appendices 29, 30).

Fixed-effect meta-analyses were performed to determine the robustness of the conclusions. These showed a nonsignificant effect of exon skipping on 6MWT and NSAA results at week 24 (Additional file 1: Appendices 31, 32).

Studies by Mendell 2013 [23] and Voit 2014 [20] were considered to have a high risk of bias in terms of selective reporting (Fig. 2). Sensitivity analysis excluding these two studies did not show significant differences in the 6MWT distance measured at 24 weeks (Additional file 1: Appendix 33).

Due to the small number of reports considered, publication bias based on funnel plot asymmetry could not be assessed. The summary of findings evaluating the quality of evidence is shown in Table 2.

**Table 2:** Summary of findings for the main comparisons

Summary of findings:					
Exon skipping compared to placebo for Duchenne muscular dystrophy					
Patient or population: Duchenne muscular dystrophy					
Setting:					
Intervention: Exon skipping					
Comparison: Placebo					
OutcomeNo of participants(studies)	Relative effect(95% CI)	Anticipated absolute effects (95% CI)			Certainty
		Without Exon skipping	With Exon skipping	Difference	
6MWT at week 24 (change from baseline,metres)№ of participants: 291(4 RCTs)	–	The mean 6MWT at week 24 (change from baseline,metres) ranged from −29.11 to −3.6 m	–	MD 9.16 m lower(21.94 lower to 3.62 higher)	⨁⨁◯◯LOW ^a,b,c,d^
NSAA at week24 (change from baseline)№ of participants: 116(3 RCTs)	–	The mean NSAA at week24 (change from baseline) ranged from −0.6 to 3.3	–	MD 1.2 higher(2.35 lower to 4.75 higher)	⨁◯◯◯VERY LOW ^a,b,c,d,e,f,g,h,i,j^
Adverse events: injection site reaction№ of participants: 322(5 RCTs)	RR 2.54(0.95 to 6.81)	14.4%	36.6%(13.6 to 98.2)	22.2% more(0.7 fewer to 83.8 more)	⨁◯◯◯VERY LOW ^a,b,c,d,f,h^
Adverse events: renal toxicity№ of participants: 322(5 RCTs)	RR 1.72(1.07 to 2.78)	16.3%	28.1%(17.5 to 45.4)	11.8% more(1.1 more to 29.1 more)	⨁◯◯◯VERY LOW ^a,b,c,d,i,j^
6MWT at week 48 (change from baseline,meters)№ of participants: 226(2 RCTs)	–	The mean 6MWT at week 48 (change from baseline,meters) ranged from −52.65 to −24.7	–	MD 19.11 lower(39.88 lower to 1.66 higher)	⨁⨁◯◯LOW ^a,b,c,d^
Timed test: 4stair climb at week 24 (change from baseline, seconds)№ of participants: 116(3 RCTs)	–	The mean timed test: 4stair climb at week 24 (change from baseline, seconds) ranged from −1.22 to 0.59 s	–	MD 0.17 s lower(0.97 lower to 0.62 higher)	⨁◯◯◯VERY LOW ^a,b,c,d,f^
QOL at week 48 (PedsQL),patient (change from baseline)№ of participants: 219(2 RCTs)	–	The mean QOL at week 48 (PedsQL),patient (change from baseline) ranged from 0.37 to 0.52	–	MD 1.53 lower(4.69 lower to 1.63 higher)	⨁⨁◯◯LOW ^a,b,c,d^

*The risk in the intervention group (and its 95% confidence interval) is based on the assumed risk in the comparison group and the **relative effect** of the intervention (and its 95% CI)

**CI:** Confidence interval; **MD:** Mean difference; **RR:** Risk ratio

**GRADE Working Group grades of evidence**

**High certainty:** We are very confident that the true effect lies close to that of the estimate of the effect

**Moderate certainty:** We are moderately confident in the effect estimate: The true effect is likely to be close to the estimate of the effect, but there is a possibility that it is substantially different

**Low certainty:** Our confidence in the effect estimate is limited: The true effect may be substantially different from the estimate of the effect

**Very low certainty:** We have very little confidence in the effect estimate: The true effect is likely to be substantially different from the estimate of effect

## Discussion

To the best of our knowledge, this is the first systematic review and meta-analysis evaluating evidence for the efficacy of exon-skipping drugs for DMD by combining all available studies. The results of our meta-analysis showed no significant overall effect of exon-skipping treatment. Subgroup analysis of the once-weekly 6 mg/kg injection of drisapersen showed significant improvement in the 6MWT in the drisapersen group. However, this data should be interpreted with caution because the risk of a type 1 error may have increased because of multiple simultaneous statistical analyses being performed. The significant effect of 6 mg/kg/ injection of drisapersen may be a false positive finding.

Subgroup analysis also revealed no significant side effects with eteplirsen treatment, but a substantial number of participants receiving drisapersen suffered from injection site reactions and renal toxicity. This difference in side effects may be due to the difference in the chemical structures of the two compounds. Drisapersen employs 2’O-methyl-phosphorothioate oligonucleotides (2’OMePS) with negatively charged phosphorothioate internucleotide linkages. In contrast, eteplirsen is based on phosphorodiamidate morpholino oligomers, which are neutrally charged. The neutrally charged nature of eteplirsen is reported to reduce off-target effects and immune response [25].

As there was only one RCT evaluating eteplirsen, the question as to whether it positively changes the clinical course of DMD or has any significant side effects remains open. Concerns about the efficacy and safety of drisapersen had already been noted prior to our review; in recognition of that fact, the U.S. FDA refrained from approving drisapersen in 2015 [11]. Despite possible effectiveness when used at a specific dosage, this meta-analysis has added methodological and statistical evidence to our current understanding over the effects of drisapersen.

Two out of the five RCTs were judged as having a high risk of bias. Specifically, Mendell 2013 [23] did not report on all the datasets that were pre-specified in the methods, such as a change in QOL scores, which was identified as an important outcome in our study based on the opinions of both patients and physicians. There was a high risk of bias in group similarity in Mendell 2013 [23] due to the fact that a sibling pair with clinically rapid deterioration was allocated to the exon skipping group. This might have driven the study results in favor of the placebo, thereby concealing any potential efficacy of eteplirsen. The trial by Voit 2014 [20] was also considered to be at high risk of reporting bias due to limited availability of pre-specified outcomes. Taken together, our analysis has uncovered certain technical drawbacks in the results of published clinical trials of exon skipping drugs that suggest possible erroneous interpretation of drug effects.

The European Union regulatory bodies have indicated the necessity for stratification according to GCs use to avoid confounding by variation in supportive measures [26]. However, none of the studies we assessed analyzed their results based on concomitant usage of GCs, nor provided any data on their use. Hence, a subgroup analysis based on GC use was not feasible, so that the treatment effects seen in patients taking exon skipping drugs could not be identified as the result of those drugs alone.

Three out of five studies had pre-specified the 6MWT as a primary endpoint (Table 1). While our analysis showed no significant heterogeneity in the 6MWT results among the drisapersen-treated groups, results of both NSAA and the time taken to walk 10 m had substantial or considerable heterogeneity, respectively. This result is in agreement with a previous report that demonstrated higher test-retest reliability for the 6MWT compared with the NSAA or the 10-m walk test [27], implying that of the currently available measures, the 6MWT may be a more reliable outcome variable. However, whether the 6MWT can adequately represent disease progression or describe the changes in the clinical course in DMD is unclear. There is no defined set of required or recommended outcome measures for clinical trials in DMD [26, 28], and outcome measures that are adequately sensitive to true changes in disease course in these patients still need to be identified.

During the protocol development of the current systematic review, survival was identified as an important outcome, yet none of the studies has provided relevant data. Studies with an endpoint such as survival would require long-term observation, which may be possible in a post-approval situation [26]. Long-term data need to be collected to understand the effects of the drug on survival.

Moderate to substantial heterogeneity in the 6MWT results and in AEs were observed between the eteplirsen and the drisapersen groups, indicating diversity in mechanism of action between these two agents. The therapeutic potential of such AOs have been proven in other genetic disorders, for example, nusinersen for spinal muscular atrophy type 1 [29]. Our analysis suggests that exon skipping drugs with different chemical structures, routes of administration, or targeted sequences may have different effects on the disease. In addition, all five studies are about skipping exon 51 because exon 51 skipping can treat the largest subset of DMD patients and molecular patches targeting exon 51 were the first to be clinically developed [8]. Studies on exon skipping that target other mutation types are ongoing, but its efficacy remains an open question. Taken together, results obtained from the current meta-analysis do not conclusively negate the exon skipping approach for DMD. Nonetheless, the uncertain efficacy of eteplirsen needs to be validated, and drisapersen cannot be applied in a clinical setting unless its side effects are reduced or eliminated. An updated systematic review incorporating future clinical trials may determine whether exon skipping can positively change the clinical course in DMD.

A notable limitation of the current study is the small number of RCTs with relatively small numbers of participants. DMD is a rare condition, so the number of patients who can participate in clinical studies is limited. Long-standing critiques have been made about underpowered studies. Under such circumstances, it has been proposed that meta-analysis, especially if conducted in a prospectively planned manner, may redeem the limitations of studies with insufficient statistical power [12, 30]. A prospective meta-analysis is a collaborative research design in which individual RCTs are designed with agreed-on methods, with meta-analysis planned in advance [12, 31]. This is considered to be a useful methodology when single, large-scale trials are not feasible, and has shown success in producing evidence for cholesterol management [32] and ankylosing spondylitis [33]. Planning a prospective meta-analysis along with consideration on designing appropriate small clinical trials might be useful in future clinical trials for DMD.

Another important approach would be the use of real-world data, which is information on health care that incorporates evidence from various sources that are routinely collected in real-world settings [34]. In DMD, a recent study has used data from a global patient registry and showed greater variability in clinical progression and outcomes than previously reported, which could have masked the drug effect in the clinical trials [35]. A better understanding of the natural history of the disease may be acquired by analyzing real-world data. This analysis may contribute to the development of more precise outcomes that correspond to the disease progression or provide complementary data on clinical trials in rare diseases where it is not feasible to increase the number of participants. In such cases, the management of the quality of data collection would be indispensable.

## Conclusions

The availability of only limited data with a high risk of bias indicates the necessity of additional clinical trials using eteplirsen to clarify its effects. In fact, the FDA has mandated a post-marketing RCT to prove the efficacy of eteplirsen, with results to be reported by 2021 [11]. Planning a prospective meta-analysis of pooled data from relevant studies and the utilization of real-world data may provide a more precise estimate of the effects of exon skipping for treating DMD.

## Additional file

Additional file 1: PRISMA checklist, protocol, search term, meta-analyses on outcomes other than primary, and characteristics of excluded studies. (PDF 1341 kb)
