# Supplementary material for: Exon skipping for Duchenne muscular dystrophy: a systematic review and meta-analysis
Source: Orphanet J Rare Dis. 2018 Jun 15;13:93. doi: 10.1186/s13023-018-0834-2 (PMC6003096; doi:10.1186/s13023-018-0834-2)
Supplement: Supplementary file 1 — PRISMA checklist, protocol, search term, meta-analyses on outcomes other than primary, and characteristics of excluded studies. (PDF 1341 kb) [file 13023_2018_834_MOESM1_ESM.pdf]

Additional file 1:

PRISMA checklist, protocol, search terms, meta-analyses on outcomes other than primary, and characteristics of excluded studies

Table of Contents

Appendix 1: PRISMA check list.....3

Appendix 2: Protocol.....7

Appendix 3: Search term for CENTRAL.....25

Appendix 4: Search term for MEDLINE.....27

Appendix 5: Search term for EMBASE.....30

Appendix 6: Search term for ICHUSHI-web.....33

Appendix 7: Search terms for Primary Registries in the WHO Registry Network and in registries approved by the ICMJE.....35

Appendix 8: Adverse events (Cardiac toxicity).....36

Appendix 9: Adverse events (Gastrointestinal symptoms).....37

Appendix 10: Adverse events (Hematologic abnormality).....38

Appendix 11: Adverse events (Hemorrhage).....39

Appendix 12: Adverse events (Hepatic toxicity).....40

Appendix 13: Adverse events (Inflammation).....41

Appendix 14: Adverse events (Pain).....42

Appendix 15: Adverse events (Others).....43

Appendix 16: 6MWT at week 48 (change from baseline, meters).....44

Appendix 17: Timed test at week 24, time taken for 4 stairs climb, (change from baseline, seconds)

....45

Appendix 18: Timed test at week 24, time taken for 4 stairs descent, (change from baseline, seconds) 46

Appendix 19: Timed test at week 24, time taken for 10 minutes walk/run, (change from baseline, seconds) 47

Appendix 20: Timed test at week 24, time taken to rise from floor, (change from baseline, seconds) 48

Appendix 21: QOL (PedsQL) at week 48, parents, (change from baseline) 49

Appendix 22: QOL (PedsQL) at week 48, patient, (change from baseline)...50

Appendix 23: Number of participants who withdrew from the study 51

Appendix 24: 6MWT at week 24 (change from baseline, meters), eteplirsen vs placebo...................52

Appendix 25: 6MWT at week 24 (change from baseline, meters), drisapersen vs placebo................53

Appendix 26: NSAA at week 24 (change from baseline), eteplirsen vs placebo 54

Appendix 27: NSAA at week 24 (change from baseline), drisapersen vs placebo...............................55

Appendix 28: 6MWT at week 24 , (change from baseline, meters), drisapersen 6mg/kg/time weekly injection..56

Appendix 29: Adverse events (Injection site reaction, drisapersen 6mg/kg/time weekly injection)..57

Appendix 30: Adverse events (Renal toxicity, drisapersen 6mg/kg/time weekly injection) 58

Appendix 31: 6MWT at week 24 (change from baseline, meters), fixed-effect meta-analysis...........59

Appendix 32: NSAA at week 24 (change from baseline), fixed-effect meta-analysis 60

Appendix 33: 6MWT at week 24 (change from baseline, meters), excluding Mendell 2013 and Voit 2014 which were considered to possess high risk of bias..61

Appendix table 1: Characteristics of excluded studies...62

Appendix 1: PRISMA check list

Section/topic	#	Checklist item	Reported on page #
**TITLE**			
Title	1	Identify the report as a systematic review, meta-analysis, or both.	1
**ABSTRACT**			
Structured summary	2	Provide a structured summary including, as applicable: background; objectives; data sources; study eligibility criteria, participants, and interventions; study appraisal and synthesis methods; results; limitations; conclusions and implications of key findings; systematic review registration number.	3-4
**INTRODUCTION**			
Rationale	3	Describe the rationale for the review in the context of what is already known.	6
Objectives	4	Provide an explicit statement of questions being addressed with reference to participants, interventions, comparisons, outcomes, and study design (PICOS).	7
**METHODS**			
Protocol and registration	5	Indicate if a review protocol exists, if and where it can be accessed (e.g., Web address), and, if available, provide registration information including registration number.	7
Eligibility criteria	6	Specify study characteristics (e.g., PICOS, length of follow-up) and report characteristics (e.g., years considered, language, publication status) used as criteria for eligibility, giving rationale.	8
Information sources	7	Describe all information sources (e.g., databases with dates of coverage, contact with study authors to identify additional studies) in the search and date last searched.	8-9

Search	8	Present full electronic search strategy for at least one database, including any limits used, such that it could be repeated.	Appendix 1
Study selection	9	State the process for selecting studies (i.e., screening, eligibility, included in systematic review, and, if applicable, included in the meta-analysis).	9
Data collection process	10	Describe method of data extraction from reports (e.g., piloted forms, independently, in duplicate) and any processes for obtaining and confirming data from investigators.	9
Data items	11	List and define all variables for which data were sought (e.g., PICOS, funding sources) and any assumptions and simplifications made.	9
Risk of bias in individual studies	12	Describe methods used for assessing risk of bias of individual studies (including specification of whether this was done at the study or outcome level), and how this information is to be used in any data synthesis.	10
Summary measures	13	State the principal summary measures (e.g., risk ratio, difference in means).	10
Synthesis of results	14	Describe the methods of handling data and combining results of studies, if done, including measures of consistency (e.g., I^2) for each meta-analysis.	11

**Section/topic**	**#**	**Checklist item**	**Reported on page #**
Risk of bias across studies	15	Specify any assessment of risk of bias that may affect the cumulative evidence (e.g., publication bias, selective reporting within studies).	10

Additional analyses	16	Describe methods of additional analyses (e.g., sensitivity or subgroup analyses, meta-regression), if done, indicating which were pre-specified.	10-11
**RESULTS**			
Study selection	17	Give numbers of studies screened, assessed for eligibility, and included in the review, with reasons for exclusions at each stage, ideally with a flow diagram.	11-12
Study characteristics	18	For each study, present characteristics for which data were extracted (e.g., study size, PICOS, follow-up period) and provide the citations.	Table1
Risk of bias within studies	19	Present data on risk of bias of each study and, if available, any outcome level assessment (see item 12).	12-13
Results of individual studies	20	For all outcomes considered (benefits or harms), present, for each study: (a) simple summary data for each intervention group (b) effect estimates and confidence intervals, ideally with a forest plot.	13-16
Synthesis of results	21	Present results of each meta-analysis done, including confidence intervals and measures of consistency.	13-16
Risk of bias across studies	22	Present results of any assessment of risk of bias across studies (see Item 15).	12-13
Additional analysis	23	Give results of additional analyses, if done (e.g., sensitivity or subgroup analyses, meta-regression [see Item 16]).	14-15
**DISCUSSION**			
Summary of evidence	24	Summarize the main findings including the strength of evidence for each main outcome; consider their relevance to key groups (e.g., healthcare providers, users, and policy makers).	Table2
Limitations	25	Discuss limitations at study and outcome level (e.g., risk of bias), and at review-level (e.g., incomplete retrieval of identified research, reporting bias).	20-21

Conclusions	26	Provide a general interpretation of the results in the context of other evidence, and implications for future research.	21
**FUNDING**			
Funding	27	Describe sources of funding for the systematic review and other support (e.g., supply of data); role of funders for the systematic review.	25

From: Moher D, Liberati A, Tetzlaff J, Altman DG, The PRISMA Group (2009). Preferred Reporting Items for Systematic Reviews and Meta-Analyses: The PRISMA Statement. PLoS Med 6(7): e1000097. doi:10.1371/journal.pmed1000097

For more information, visit: **www.prisma-statement.org**.

Page 2 of 2

Appendix 2: Protocol

Exon skipping for Duchenne Muscular Dystrophy (Protocol)

Yuko Shimizu-Motohashi, MD[1], Terumi Murakami, MD, Ph.D[2], En Kimura, MD, Ph.D[3], Hirofumi Komaki, MD, Ph.D[1], Norio Watanabe*, MD, Ph.D[4]

[1] Department of Child Neurology, National Center Hospital, National Center of Neurology and Psychiatry, Tokyo, Japan.

[2] Department of Neurology, Higashisaitama National Hospital, 4147 Kurohama, Hasuda, Saitama 349-0196, Japan.

[3] Translational Medical Center, National Institute of Neuroscience, National Center of Neurology and Psychiatry, Tokyo, Japan.

[4] Department of health Promotion and Human Behavior, Graduate School of Medicine/ School of Public Health, Kyoto University, Kyoto, Japan.

* Address correspondence to: Norio Watanabe, MD, Ph.D.

Department of health Promotion and Human Behavior, Graduate School of Medicine/ School of Public Health, Kyoto University, Kyoto, Japan

Tel.: +81-75-753-9491, Fax: +81-75-753-4641

noriowncu@gmail.com

ABSTRACT

This is the protocol for a review and there is no abstract. The objectives are as follows:

Objective

To assess whether exon skipping can positively change the clinical course of DMD patients.

BACKGROUND

Description of the condition

Duchenne muscular dystrophy (DMD) is caused by the mutation in the *DMD* located on Xp21[1], which affects predominantly male individuals. It is the most common hereditary muscular disorder, estimated to affect one in 3500-6000 live male birth [2-4]. The protein product of the *DMD*, dystrophin, links the muscle sarcomeric structure to the extracellular matrix by constructing dystrophin–associated glycoprotein complex (DGC) [5] which confers to the strength of sarcolemma [6]. Individuals with DMD have absent or decreased level of dystrophin, manifesting walking difficulty at around age of five, and they become wheel chair bound before or during their teens [7, 8]. The distance of which a subject is able to walk in six minutes assessed by six-minute walk test (6MWT), is interpreted as the measure of physical capacity and walking function [9, 10]. In DMD, the longitudinal observational study had shown an increase in the distance of 6MWT until age of 7.5 years, followed by a decline, which became precipitous from 12.5 years to 15.5 years, when all boys were unable to perform the 6MWT [11]. Along with the motor function decline, patients also show respiratory and cardiac dysfunction owing to loss of respiratory muscle strength and cardiac tissue degeneration [12]. Preclinical cardiomyopathy becomes apparent in patients less than age 6 years [13], and respiratory function peaks out before age of 12 [14]. Without supportive care, patients typically die in their late teens or in early 20s [15]. Intervention with mechanical ventilation and cardioprotective medications have improved the survival, and patients may live to their 30s [16].

Currently, there is no curative therapy for DMD and glucocorticoids are the only medication available that slows the decline in muscle strength and function [7]. The clinical efficacy of glucocorticoids is established and is considered to be the standard therapy for patients with DMD, however, there are several concerns over the glucocorticoid therapy. One is that glucocorticoids have serious side effects in long-term usage, including weight gain, cushignoid, behavior change, growth delay, cataracts and osteoporosis [17]; second, the mechanism how glucocorticoids contribute to beneficial effects in patients with DMD is still unknown. Owing to the myriads of side effects, not all patients are eligible for receiving or continuing the glucocorticoid therapy.

Development of innovative therapies for patients with DMD is an urgent task, and several therapeutic approaches are now being investigated, including exon skipping, read through, vector-mediated gene therapy, cell transplantation, anti-inflammatory/fibrotic/oxidant drugs, myostatin pathway inhibitors, nNOS pathway enhancement, and utrophin up-regulation [18, 19]. Amongst all these approaches, exon skipping, read through and antioxidants are at the most advanced stage of clinical trials. Two major drugs evaluated for exon skipping are Drisapersen (BioMarin Pharmaceutical Inc.) and eteplirsen (Sarepta

Therapeutics), and these have been either completed or now being studied in phase 3 clinical trials (https://clinicaltrials.gov/). Ataluren, the compound for read through has also completed phase 3 study and now has an approval from European Medicines Agency (EMA) for marketing in Europe [20]. Idebenone, an antioxidant has also completed phase 3 trial [21]. Exon skipping and read through aim to treat the disease by restoring dystrophin, whereas idebenone aims to compensate for the lack of dystrophin. Each therapeutic approach bears pros and cons in their nature. Dystrophin is expressed not only in skeletal muscle but also in brain, and have been reported that significant proportion of DMD patients have cognitive and learning disabilities [22]. From the viewpoint that DMD is not only confined to the skeletal muscle but more of a systemic disorder, exon skipping or read through may have more benefit than idebenone.

The idea of exon skipping approach for DMD have emerged in 1990s [23], based on the rationale of converting the translational reading frame from out-of-frame to in-frame to produce shorter but functional dystrophin, instead of degradable dystrophin. Exon skipping can be applicable to versatile mutation types including deletion, duplication, and small mutations, theoretically applicable to up to 83% of total mutation [24], whereas read-through can treat only patients with nonsense mutation which comprises 10% of total mutation [25].

Although it is yet to be clarified whether the efficacy of exon skipping can exceed that of glucocorticoids in DMD patients, exon skipping is one of the most promising approach, with scientific rationale and accumulation of i*n vitro*[26-28] and *in vivo*[29-31] study data. However, clinical trials for exon skipping have failed to demonstrate statistically significant improvement in motor function so far [32, 33]. Several factors can be thought for the reasons why it is so difficult to replicate pre-clinical research data in patients. One may be the low prevalence of DMD, and that it interferes with designing a large cohort study with sufficient power to detect the efficacy. Hence, a meta-analysis is necessary to assess the efficacy of exon skipping for patients with DMD.

To date, the results from multiple clinical trials have not been reviewed in a standardized manner. Herein, we propose to conduct a systematic review and meta-analysis on exon skipping studies in DMD to assess the potential and the limitation of exon skipping.

Description of the intervention

Exon skipping

Exon skipping induced by antisense oligonucleotides (AOs) modulates dystrophin pre-mRNA splicing process and restores the reading frame of *DMD* [34]. The reading frame is converted from out-of-frame to in-frame, which generates the protein that is short but functional, instead of truncated and presumably

degradable dystrophin, and is considered to be capable of converting DMD to a milder phenotype [35]. There is a mutation hotspot in the *DMD* encoded by exons 43-45, and skipping of exon 51 can theoretically restore the largest subset of patients; therefore AOs targeting exon 51 were the first to be developed clinically [36]. There are several AO chemistries that have been investigated of which 2'O-methyl-phosphorothioate oligonucleotide (2'OMePS) and phosphorodiamidate morpholino oligomer (PMO) are the two major drug candidates that are currently being evaluated in advanced phase of clinical trials.

2'O-methyl-phosphorothioate oligonucleotide (2'OMePS)

The 2'OMePS chemistry has internucleotide phosphorothioate linkages, and are negatively charged. The chemistry targeting exon 51 have completed phase 3 clinical trial, and phase 1 or 2 studies targeting exon 44, 45, 53 are now being conducted (https://clinicaltrials.gov/).

Phosphorodiamidate morpholino oligomer (PMO)

The PMO chemistry is a charge-neutral compound, and have reduced off-target effects, along with less immunoreactivity [37]. Phase 3 clinical trial is now being conducted for exon 51 skipping, along with phase 1 or 2 study for exon 45 and 53 skipping (https://clinicaltrials.gov/).

Others

The chemistries that have been investigated other than 2'OMePS and PMOs are 2'-O,4'-C-Ethylene-bridged Nucleic Acids (ENA), locked nucleic acid (LNA), peptide nucleic acid (PNA), tricyclo0DNA (tcDNA), cell-penetrating peptide-conjugated phosphorodiamidate morpholino oligomers (PPMOs), arginine-rich cell-penetrating peptide-conjugated PMOs (Pip-PMOs), and vivo-morpholinos (vPMOs) [34, 37]. LNA-modified AOs display better mismatch discrimination and high resistance against nucleases; tcDNA show enhanced target-binding affinity and improved nuclease resistance [34]; PPMOS, Pip-PMOs, vPMOs contain cell-penetrating moieties [37]. Viral vectors and synthetic vectors (liposomes, cationic peptides and polymers, protein complexes) and covalent attachment such as antibodies, peptides, lipids, carbohydrates, growth factors and vitamins have been tried to overcome the poor target-specific delivery [34] and relatively unstable skipping efficiency of AOs, which are the current hurdles of exon skipping strategy.

Drugs which improve the underlying dystrophic condition

Glucocorticoids including;

- Prednisolone

- Prednisone
- Deflazacort
- Any other glucocorticoids, if identified

Why it is important to do this review

Currently there is no curative therapy for DMD patients. Exon skipping is a therapeutic approach that have been investigated for decades which now have become the strategy at the most advanced phase of clinical trials. Review of the results from clinical studies is crucial for understanding the efficacy and the limitation of exon skipping.

OBJECTIVES

To assess whether exon skipping can positively change the clinical course of patients with DMD.

METHODS

The systematic review of the literature will comply with the PRISMA statement.

Criteria for considering studies for this review

Types of studies

We will include double-blind, randomized controlled trials (RCTs) that investigate the effect of exon skipping in patients with DMD. We will also include the first phase of cross-over controlled trials. Comparison will be done between: the effects of exon skipping and placebo, both groups without glucocorticoid therapy; the effects of exon skipping and placebo, both groups with continuous glucocorticoid therapy; the effects of exon skipping and placebo, both groups with intermittent glucocorticoid therapy.

Types of participants

All patients, including children and adults of all ages, gender, race, out and inpatients, who are confirmed to have out-of-frame *DMD* mutations that are identified by the authors as correctable by exon skipping are eligible for the review. The genetic analysis must be confirmed by any of the published or author approved methodologies that evaluate all *DMD* exons, including multiplex ligation-dependent probe, comparative genomic hybridization, single condition amplification/internal primer analysis, and target resequencing.

Studies with participants who are receiving steroid therapies are eligible for this meta-analysis, but the

participants must be on steroid before the first administration of exon skipping drug.

Types of interventions

Exon skipping chemistries that will be reviewed are:

- 2'OMePS
    - Drisapersen
    - PRO044
    - PRO045
    - PRO053
- PMO
    - Eteplirsen
    - SRP-4045
    - SRP-4053
    - NS-065/NCNP-01
- Any other exon skipping chemistry if identified.

Administration plan that will be reviewed are:

- Subcutaneous, intravenous injection, and any other administration route if identified.
- Single dose, short and long term administration, and any other administration period if identified.
- Dosage ranging from low dose to high dose, and any other dosage if identified. Our preliminary investigation of previous reports[32, 38-40] suggested as below:
    - For drisapersen subcutaneous injection, the dose range will be defined as low for <5mg/kg/ time, moderate for 5-10mg/kg/ time and high for >10mg/kg/ time
    - For drisapersen intravenous injection, the dose range will be defined as low for <1mg/kg/ time, moderate for 1-2mg/kg/ time and high for >2mg/kg/ time.
    - For eteplirsen, the dose range will be defined as low for <20mg/kg/time, moderate for 20-40mg/kg/ time and high for >40mg/kg/ time
- Intermittent or continuous administration, and any other administration schedule if any identified.
- Both out and inpatient administration.
- Placebo include mannitol, phosphate-buffered saline (PBS), or any other placebo if identified.
- Steroid therapies will be accepted if both treated and control groups are receiving steroid.

●

We plan to analyze data for each type of intervention separately.

Type of outcome measures

The outcomes listed here are not eligibility criteria for this review, but are outcomes of interest within whichever studies are included.

Primary outcome

We will assess the data of change in six-minute walk test (6MWT) distance from the baseline obtained between 20-35 weeks after the treatment initiation as the primary outcome, and will refer it as “data of week 24”. If there are multiple data collected within the above designated weeks, than the data obtained at the week closest to week 24 will be selected for the review.

Six-minute walk test (6MWT) has been used to assess functional capacity in patients with heart and lung related diseases, and it was the primary endpoint in a study that supported regulatory approval of drug for primary pulmonary hypertension [41]. More recently, its relevance to neuromuscular diseases including DMD has been established [42].

For the participants who became non-ambulatory during the study, the 6MWT data will be recorded as 0.

Secondary outcomes

- Change in 6MWT distance from the baseline evaluated outside the time-line defined for the primary outcome measurement.
    - data obtained between 2-7 weeks after the treatment initiation will be represented as “ data of week 4”
    - data obtained between 8-19 weeks after the treatment initiation will be represented as “ data of week 12”
    - data obtained between 36-52 weeks after the treatment initiation will be represented as “ data of week 48”
    - If there were multiple data collected within the above designated weeks, than the data obtained at the week closest to the representative weeks (i.e., week 4, 12, 48 after the treatment initiation) will be selected for the review.
    - Any other weeks, if identified
- The percentage of dystrophin positive fibers.
- Pulmonary function

    - Forced Vital Capacity (FVC), forced Expiratory Volume (FEV1), Maximum inspiratory/expiratory pressure percent predicted (MIP/MEP % predicted)
- Muscle function
    - North Star Ambulatory Assessment (NSAA) [43], timed tests (10-meter walk/run, rising from floor, stair climb) [44], DMD Functional Outcomes Questionnaire (DMD-FOS), Egen Klassification [45], Performance Upper Limb (PUL) [46], Patient Reported Outcome measure (PROM)
- Muscle strength
    - Quantitative Muscle Testing (QMT), Manual Muscle Testing (MMT), handheld myometry
- Cardiac function
    - Electrocardiography (QT interval, rhythm)
    - Echocardiography (Ejection fraction, Fraction shortening)
- Production of exon skipped mRNA in muscle biopsy
- Serum creatine kinase level
- Number of patients who dropped out from the protocol
- Number of patients who deviated from the protocol
- Number of patients who discontinued the protocol
- Number of patients who stopped to be assessed
- Adverse events
    - Injection site reaction, inflammation, gastrointestinal symptoms, hemorrhage, renal/ hepatic/ cardiac toxicity, hematologic abnormality, pain, and any other adverse events if identified.
- Pharmacokinetics
    - T1/2, Cmax, Ctrough, 7d, tmax,, volume of distribution, clearance
- Change in the quality of life (QoL) of the individuals who received exons skipping, as well as their caregivers.
- Change in the ability of daily life (ADL) of the individuals who received exon skipping.
- Change in the amount of care provided by the carergivers of the individuals who received exon skipping.
- Change in the mental health status of the individuals who recieved exon skipping as well as their caregivers.
- Survival

Search methods for identification of studies

Electronic searches

We will search the the Cochrane Central Register of Controlled Trials (CENTRAL), MEDLINE via Ovid (from January 1990 to the present), EMBASE via embase.com (from January 1990 to the present), ICHUSHI-web (Japana Centra Revuo Medicina), Clinical trials.gov, FDA website, EMEA website, any other clinical trial registries if identified.

The details of search strategies are shown in Appendix (CENTRAL), Appendix (MEDLINE), Appendix (EMBASE), and Appendix (ICHUSHI-web).

We will not limit our search by language or publication status. We will search both peer reviewed and non-peer reviewed publications. Studies reporting results will be searched in all Primary Registries in the WHO Registry Network and in registries approved by the International Committee of Medical Journal Editors (ICMJE), that meet the requirements of the ICMJE (WHO, 2013). We will also search any other registries, if any identified.

Searching other resources

We will review conference proceedings for non-published studies. We will screen bibliographies of identified manuscripts for studies not identified by the search. For large clinical trials sponsored by pharmaceutical companies known to the group, but does not appear in any of the electronically searched sites, the information will be retrieved from the pharamceutical companies' websites or will be directly contacted. We will also contact the researchers who are conducting RCTs of exon skipping for information on other exon skipping RCTs.

Data collection and analysis

Two authors will independently review the titles and abstracts identified from the register and will determine for eligibility. Two authors will obtain the full text of all potentially relevant studies for independent assessment. Two authors will independently decide which trials fit the inclusion criteria. Authors will resolve disagreements about inclusion criteria by discussion, to reach a consensus. If persistent, the disagreement will be resolved by a third author.The authorships of the studies will not be blinded prior to the assessment.

Selection of studies

We will select only randomized controlled trials, and cross-over trials for inclusion. In the Discussion, we

will review open studies, longitudinal observational studies and individual case reports but will only discuss studies in which the diagnosis, intervention, pre-treatment and post-treatment states are adequately described and in whom follow up for at least six months is available.

Data extraction and management

Two review authors will independently extract data onto pre-agreed data extraction forms. We will resolve disagreements by discussion with the other authors. One author will enter data into the Cochrane statistical software RevMan (Review Manager (RevMan) [Computer program]. Version 5.3. Copenhagen: The Nordic Cochrane Centre, The Cochrane Collaboration, 2014.) and a second author will check data entry. We will contact trial authors directly for any missing data.

Assessment of risk of bias in included studies

Two authors will independently assess included studies for risk of bias using pre-agreed criteria, described in the *Cochrane Handbook for Systematic Reviews of Interventions* (http://handbook.cochrane.org/). We will resolve disagreements by discussion with the other authors. We will grade each aspect for risk of bias as high risk ('No'), uncertain ('Unclear') or low risk ('Yes') for the following domains:

• sequence generation;
• allocation concealment;
• blinding of participants;
• blinding of outcome assessors;
• incomplete outcome data;
• selective outcome reporting; and
• other sources of bias.

Measures of treatment effect

Statistical methods used to measure treatment effects will be in accordance with The *Cochrane Handbook for Systematic Reviews of Interventions* (http://handbook.cochrane.org/).

We will analyze dichotomous data as risk ratios (RR) and continuous data as mean difference, or standardized mean difference for results across studies with outcomes that are conceptually the same but measured in different ways. We will report these measures of effect with 95% confidence intervals (CI).

We will undertake meta-analyses, using RevMan, only where this is meaningful, that is if the treatments, participants and the underlying clinical question are similar enough for pooling to make sense.

Unit of analysis issues

Where a single trial includes multiple trial arms, we will include only the relevant arms. If we combine two comparisons (e.g. drug A versus placebo and drug B versus placebo) in the same meta-analysis, we will halve the control group to avoid double-counting. For each participant there may be multiple observations for the same outcome.

Dealing with missing data

If necessary, we will attempt to contact trial authors for missing data including numbers of dropouts and deaths and whether or not an intention-to-treat analysis was performed.

Assessment of heterogeneity

We will assess clinical heterogeneity by judging, qualitatively, the differences between studies regarding the participants, therapies, and reporting of important study outcomes.

We will statistically test heterogeneity of intervention effects among trials using the standard Chi^2 statistic (P value) and the Higgins I^2 statistic expressed as a percentage. We will take P values of less than 0.05 as evidence of heterogeneity. We will interpret I^2 for heterogeneity as follows (http://handbook.cochrane.org/):

• 0% to 40%: may not be important;

• 30% to 60%: may represent moderate heterogeneity;

• 50% to 90%: may represent substantial heterogeneity;

• 75% to 100%: considerable heterogeneity.

If we identify substantial unexplained heterogeneity we will report it and explore possible causes by pre-specified subgroup analysis.

Assessment of reporting biases

To detect the presence of publication bias, we will construct a funnel plot using Revman, if there are a reasonable number of studies (at least 10 in the same meta-analysis). We will use Begg's and Egger's tests to verify the bias [47, 48].

Data synthesis

If there is no substantial or considerable heterogeneity, we will synthesize the data in a meta-analysis using RevMan. We will perform random-effects models for comparison purposes and use the most

appropriate, depending upon the degree of heterogeneity.

Subgroup analysis and investigation of heterogeneity

If there are sufficient data, we plan to undertake the following subgroup analyses using the outcome:

1. steroid combination
2. non-steroid combination.
3. different types of exon skipping drugs
4. low dose administration
5. moderate dose administration
6. high dose administration

Within each group we will use the I^2 statistic for heterogeneity and if its value is greater than 50% we will scrutinize the trials and forest plots for differences to explain the heterogeneity. If we find no explanation, we will repeat the analysis using a random-effects model.

'Summary of findings' table

We will create a 'Summary of findings' table using the following outcomes:

- Change in 6MWT;
- Change in *dystrophin* gene or protein expression; and
- Adverse events.

We will use the five GRADE considerations (study limitations, consistency of effect, imprecision, indirectness and publication bias) to assess the quality of a body of evidence (studies that contribute data for the prespecified outcomes). We will use methods and recommendations described in Section 8.5 and Chapter 12 of the *Cochrane Handbook for Systematic Reviews of Interventions* [49] using GRADEpro software. We will justify all decisions to down- or upgrade the quality of studies using foot- notes and we will make comments to aid readers' understanding of the review where necessary.

Sensitivity analysis

We will perform a sensitivity analysis to determine whether conclusions are robust by undertaking both fixed-effect and random effect meta-analysis. We will perform sensitivity analyses to assess the effect of including studies at high risk of bias on the change in 6MWT, and by repeating the meta-analysis excluding any studies at high risk of bias.

ACKNOWLEDGEMENTS

The work will be supported by The Clinical Research Program for Child Health and Development, AMED (No.27300101).

AMENDMENTS FROM THE PROTOCOL

Upon data extraction and management, the entered data into Revman software was double checked by a single author.

We planned to create a 'Summary of findings' table with outcomes including change in 6MWT, change in *dystrophin* gene or protein expression, and adverse events. However the primary outcomes for our study had changed according to physicians', patients' and their families' opinions. Therefore the 'Summary of findings' was created with outcomes including change in 6MWT after 24 weeks of treatment, change in NSAA score after 24 weeks of treatment, and adverse events.

REFERENCES

1. Monaco AP, Neve RL, Colletti-Feener C, Bertelson CJ, Kurnit DM, Kunkel LM. Isolation of candidate cDNAs for portions of the Duchenne muscular dystrophy gene. Nature. 1986; 323:646-50.
2. Mendell JR, Shilling C, Leslie ND, Flanigan KM, al-Dahhak R, Gastier-Foster J et al. Evidence-based path to newborn screening for Duchenne muscular dystrophy. Annals of neurology. 2012; 71:304-13.
3. Moat SJ, Bradley DM, Salmon R, Clarke A, Hartley L. Newborn bloodspot screening for Duchenne muscular dystrophy: 21 years experience in Wales (UK). Eur J Hum Genet. 2013; 21:1049-53.
4. Emery AE. Population frequencies of inherited neuromuscular diseases--a world survey. Neuromuscul Disord. 1991; 1:19-29.
5. Ervasti JM, Ohlendieck K, Kahl SD, Gaver MG, Campbell KP. Deficiency of a glycoprotein component of the dystrophin complex in dystrophic muscle. Nature. 1990; 345:315-9.
6. Zubrzycka-Gaarn EE, Bulman DE, Karpati G, Burghes AH, Belfall B, Klamut HJ et al. The Duchenne muscular dystrophy gene product is localized in sarcolemma of human skeletal muscle. Nature. 1988; 333:466-9.

7. Bushby K, Finkel R, Birnkrant DJ, Case LE, Clemens PR, Cripe L et al. Diagnosis and management of Duchenne muscular dystrophy, part 1: diagnosis, and pharmacological and psychosocial management. Lancet Neurol. 2010; 9:77-93.
8. Bushby KM, Hill A, Steele JG. Failure of early diagnosis in symptomatic Duchenne muscular dystrophy. Lancet (London, England). 1999; 353:557-8.
9. ATS statement: guidelines for the six-minute walk test. Am J Respir Crit Care Med. 2002; 166:111-7.
10. Goemans N, Klingels K, van den Hauwe M, Boons S, Verstraete L, Peeters C et al. Six-minute walk test: reference values and prediction equation in healthy boys aged 5 to 12 years. PloS one. 2013; 8:e84120.
11. Goemans N, van den Hauwe M, Wilson R, van Impe A, Klingels K, Buyse G. Ambulatory capacity and disease progression as measured by the 6-minute-walk-distance in Duchenne muscular dystrophy subjects on daily corticosteroids. Neuromuscul Disord. 2013; 23:618-23.
12. Bushby K, Finkel R, Birnkrant DJ, Case LE, Clemens PR, Cripe L et al. Diagnosis and management of Duchenne muscular dystrophy, part 2: implementation of multidisciplinary care. Lancet Neurol. 2010; 9:177-89.
13. Nigro G, Comi LI, Politano L, Bain RJ. The incidence and evolution of cardiomyopathy in Duchenne muscular dystrophy. Int J Cardiol. 1990; 26:271-7.
14. Finder JD, Birnkrant D, Carl J, Farber HJ, Gozal D, Iannaccone ST et al. Respiratory care of the patient with Duchenne muscular dystrophy: ATS consensus statement. Am J Respir Crit Care Med. 2004; 170:456-65.
15. McNally EM, Kaltman JR, Benson DW, Canter CE, Cripe LH, Duan D et al. Contemporary cardiac issues in Duchenne muscular dystrophy. Working Group of the National Heart, Lung, and Blood Institute in collaboration with Parent Project Muscular Dystrophy. Circulation. 2015; 131:1590-8.
16. Ishikawa Y, Miura T, Ishikawa Y, Aoyagi T, Ogata H, Hamada S et al. Duchenne muscular dystrophy: survival by cardio-respiratory interventions. Neuromuscul Disord. 2011; 21:47-51.
17. Bello L, Gordish-Dressman H, Morgenroth LP, Henricson EK, Duong T, Hoffman EP et al. Prednisone/prednisolone and deflazacort regimens in the CINRG Duchenne Natural History Study. Neurology. 2015; 85:1048-55.
18. Falzarano MS, Scotton C, Passarelli C, Ferlini A. Duchenne Muscular Dystrophy: From Diagnosis to Therapy. Molecules. 2015; 20:18168-84.
19. Jarmin S, Kymalainen H, Popplewell L, Dickson G. New developments in the use of gene

therapy to treat Duchenne muscular dystrophy. Expert Opin Biol Ther. 2014; 14:209-30.

20. Mullard A. EMA reconsiders 'read-through' drug against Duchenne muscular dystrophy following appeal. Nat Biotechnol. 2014; 32:706.

21. Buyse GM, Voit T, Schara U, Straathof CS, D'Angelo MG, Bernert G et al. Efficacy of idebenone on respiratory function in patients with Duchenne muscular dystrophy not using glucocorticoids (DELOS): a double-blind randomised placebo-controlled phase 3 trial. Lancet. 2015; 385:1748-57.

22. Banihani R, Smile S, Yoon G, Dupuis A, Mosleh M, Snider A et al. Cognitive and Neurobehavioral Profile in Boys With Duchenne Muscular Dystrophy. J Child Neurol. 2015; 30:1472-82.

23. Nicholson LV. The "rescue" of dystrophin synthesis in boys with Duchenne muscular dystrophy. Neuromuscul Disord. 1993; 3:525-31.

24. Aartsma-Rus A, Fokkema I, Verschuuren J, Ginjaar I, van Deutekom J, van Ommen GJ et al. Theoretic applicability of antisense-mediated exon skipping for Duchenne muscular dystrophy mutations. Hum Mutat. 2009; 30:293-9.

25. Bladen CL, Salgado D, Monges S, Foncuberta ME, Kekou K, Kosma K et al. The TREAT-NMD DMD Global Database: analysis of more than 7,000 Duchenne muscular dystrophy mutations. Hum Mutat. 2015; 36:395-402.

26. Wilton SD, Lloyd F, Carville K, Fletcher S, Honeyman K, Agrawal S et al. Specific removal of the nonsense mutation from the mdx dystrophin mRNA using antisense oligonucleotides. Neuromuscul Disord. 1999; 9:330-8.

27. van Deutekom JC, Bremmer-Bout M, Janson AA, Ginjaar IB, Baas F, den Dunnen JT et al. Antisense-induced exon skipping restores dystrophin expression in DMD patient derived muscle cells. Hum Mol Genet. 2001; 10:1547-54.

28. Aartsma-Rus A, Janson AA, Kaman WE, Bremmer-Bout M, den Dunnen JT, Baas F et al. Therapeutic antisense-induced exon skipping in cultured muscle cells from six different DMD patients. Hum Mol Genet. 2003; 12:907-14.

29. Aoki Y, Yokota T, Nagata T, Nakamura A, Tanihata J, Saito T et al. Bodywide skipping of exons 45-55 in dystrophic mdx52 mice by systemic antisense delivery. Proceedings of the National Academy of Sciences of the United States of America. 2012; 109:13763-8.

30. Mann CJ, Honeyman K, Cheng AJ, Ly T, Lloyd F, Fletcher S et al. Antisense-induced exon skipping and synthesis of dystrophin in the mdx mouse. Proc Natl Acad Sci U S A. 2001; 98:42-7.

31. Lu QL, Mann CJ, Lou F, Bou-Gharios G, Morris GE, Xue SA et al. Functional amounts of dystrophin produced by skipping the mutated exon in the mdx dystrophic mouse. Nature medicine. 2003; 9:1009-14.
32. Mendell JR, Rodino-Klapac LR, Sahenk Z, Roush K, Bird L, Lowes LP et al. Eteplirsen for the treatment of Duchenne muscular dystrophy. Ann Neurol. 2013; 74:637-47.
33. Kole R, Krieg AM. Exon skipping therapy for Duchenne muscular dystrophy. Adv Drug Deliv Rev. 2015; 87:104-7.
34. Wilton SD, Veedu RN, Fletcher S. The emperor's new dystrophin: finding sense in the noise. Trends Mol Med. 2015; 21:417-26.
35. Shimizu-Motohashi Y, Miyatake S, Komaki H, Takeda S, Aoki Y. Recent advances in innovative therapeutic approaches for Duchenne muscular dystrophy: from discovery to clinical trials. Am J Transl Res. 2016; 8:2471-89.
36. Aartsma-Rus A, Ferlini A, Goemans N, Pasmooij AM, Wells DJ, Bushby K et al. Translational and regulatory challenges for exon skipping therapies. Hum Gene Ther. 2014; 25:885-92.
37. Echigoya Y, Yokota T. Skipping multiple exons of dystrophin transcripts using cocktail antisense oligonucleotides. Nucleic Acid Ther. 2014; 24:57-68.
38. Voit T, Topaloglu H, Straub V, Muntoni F, Deconinck N, Campion G et al. Safety and efficacy of drisapersen for the treatment of Duchenne muscular dystrophy (DEMAND II): an exploratory, randomised, placebo-controlled phase 2 study. Lancet Neurol. 2014; 13:987-96.
39. Flanigan KM, Voit T, Rosales XQ, Servais L, Kraus JE, Wardell C et al. Pharmacokinetics and safety of single doses of drisapersen in non-ambulant subjects with Duchenne muscular dystrophy: results of a double-blind randomized clinical trial. Neuromuscul Disorders. 2014; 24:16-24.
40. Cirak S, Arechavala-Gomeza V, Guglieri M, Feng L, Torelli S, Anthony K et al. Exon skipping and dystrophin restoration in patients with Duchenne muscular dystrophy after systemic phosphorodiamidate morpholino oligomer treatment: an open-label, phase 2, dose-escalation study. Lancet. 2011; 378:595-605.
41. Rubin LJ, Badesch DB, Barst RJ, Galie N, Black CM, Keogh A et al. Bosentan therapy for pulmonary arterial hypertension. N Engl J Med. 2002; 346:896-903.
42. McDonald CM, Henricson EK, Han JJ, Abresch RT, Nicorici A, Elfring GL et al. The 6-minute walk test as a new outcome measure in Duchenne muscular dystrophy. Muscle Nerve. 2010; 41:500-10.
43. Scott E, Eagle M, Mayhew A, Freeman J, Main M, Sheehan J et al. Development of a functional

assessment scale for ambulatory boys with Duchenne muscular dystrophy. Physiother Res Int. 2012; 17:101-9.

44. Brooke MH, Griggs RC, Mendell JR, Fenichel GM, Shumate JB, Pellegrino RJ. Clinical trial in Duchenne dystrophy. I. The design of the protocol. Muscle Nerve. 1981; 4:186-97.

45. Steffensen B, Hyde S, Lyager S, Mattsson E. Validity of the EK scale: a functional assessment of non-ambulatory individuals with Duchenne muscular dystrophy or spinal muscular atrophy. Physiother Res Int. 2001; 6:119-34.

46. Mayhew A, Mazzone ES, Eagle M, Duong T, Ash M, Decostre V et al. Development of the Performance of the Upper Limb module for Duchenne muscular dystrophy. Dev Med Child Neurol. 2013; 55:1038-45.

47. Begg CB, Mazumdar M. Operating characteristics of a rank correlation test for publication bias. Biometrics. 1994; 50:1088-101.

48. Egger M, Davey Smith G, Schneider M, Minder C. Bias in meta-analysis detected by a simple, graphical test. BMJ. 1997; 315:629-34.

49. Higgins J, Green S. Cochrane Handbook for Systematic Reviews of Interventions Version 5.1.0 [updated March 2011]. The Cochrane Collaboration, 2011 Available from http://www.handbook.cochrane.org. .

Appendix 3: Search term for CENTRAL

#1 MeSH descriptor: [Mu
scular Dystrophy, Duchenne] this term only

#2 MeSH descriptor: [Muscular Dystrophies] this term only

#3 (duchenne near dystroph*):ti,ab in Trials

#4 (becker near dystroph*):ti,ab in Trials

#5 dystrophinopath*:ti,ab in Trials

#6 MeSH descriptor: [Dystrophin] this term only

#7 (xldc or xldcm):ti,ab in Trials

#8 x linked dilated cardiomyopathy:ti,ab in Trials

#9 #1 or #2 or #3 or #4 or #5 or #6 or #7 or #8 in Trials

#10 MeSH descriptor: [Oligonucleotides, Antisense] explode all trees

#11 (((anti sense) or (antisense)) near oligo*):ti,ab in Trials

#12 MeSH descriptor: [Phosphorothioate Oligonucleotides] this term only

#13 phospho* oligonucleotide*:ti,ab in Trials

#14 exon* skip*:ti,ab in Trials

#15 2OME*:ti,ab in Trials

#16 "2O ME*":ti,ab in Trials

#17 "2 OME*":ti,ab in Trials

#18 drisapersen:ti,ab in Trials

#19 PRO044:ti,ab in Trials

#20 BMN044:ti,ab in Trials

#21 PRO045:ti,ab in Trials

#22 BMN045:ti,ab in Trials

#23 PRO051:ti,ab in Trials

#24 BMN051:ti,ab in Trials

#25 Kyndrisa:ti,ab in Trials

#26 PRO053:ti,ab in Trials

#27 BMN053:ti,ab in Trials

#28 phosphorodiamidate morpholino oligomer*:ti,ab in Trials

#29 PMO*:ti,ab in Trials

#30 eteplirsen:ti,ab in Trials

#31 SRP-4045:ti,ab in Trials

#32 SRP-4053:ti,ab in Trials

#33 AVI-4658:ti,ab in Trials

#34 NS-065*:ti,ab in Trials

#35 NCNP-01*:ti,ab in Trials

#36 AON:ti,ab in Trials

#37 AONs:ti,ab in Trials

#38 AO:ti,ab in Trials

#39 Aos:ti,ab in Trials

#40 Ethylene bridged Nucleic Acid*:ti,ab in Trials

#41 ENA*:ti,ab in Trials

#42 DS 5141b:ti,ab in Trials

#43 locked nucleic acid*:ti,ab in Trials

#44 LNA*:ti,ab in Trials

#45 MeSH descriptor: [Peptide Nucleic Acids] this term only

#46 peptide nucleic acid*:ti,ab in Trials

#47 PNA*:ti,ab in Trials

#48 tricyclo DNA*:ti,ab

#49 tcDNA*:ti,ab

#50 MeSH descriptor: [Cell-Penetrating Peptides] this term only

#51 cell penetrating peptide*:ti,ab in Trials

#52 vivo morpholino*:ti,ab in Trials

#53 #10 or #11 or #12 or #13 or #14 or #15 or #16 or #17 or #18 or #19 or #20 or #21 or #22 or #23 or #24 or #25 or #26 or #27 or #28 or #29 or #30 or #31 or #32 or #33 or #34 or #35 or #36 or #37 or #38 or #39 or #40 or #41 or #42 or #43 or #44 or #45 or #46 or #47 or #48 or #49 or #50 or #51 or #52 in Trials

#54 #9 and #53

Appendix 4: Search term for MEDLINE

1 Muscular Dystrophies/ or Muscular Dystrophy, Duchenne/
2 (duchenne adj5 dystroph$).tw.
3 (becker adj5 dystroph$).tw.
4 dystrophinopath$.mp.
5 Dystrophin/ge
6 (xldc or xldcm).tw.
7 x linked dilated cardiomyopathy.tw.
8 or/1-7
9 exp Oligonucleotides, Antisense/
10 ((anti sense or antisense) adj oligo$).tw.
11 Phosphorothioate Oligonucleotides/
12 phospho$ oligonucleotide$.tw.
13 (exon* and skip*).tw.
14 2OME*.tw.
15 '2' O ME*.tw.
16 '2O ME*'.tw.
17 '2' OME*.tw.
18 drisapersen.tw.
19 PRO044.tw.
20 BMN044.tw.
21 PRO045.tw.
22 BMN045.tw.
23 PRO051.tw.
24 BMN051.tw.
25 Kyndrisa.tw.
26 PRO053.tw.
27 BMN053.tw.
28 phosphorodiamidate morpholino oligomer$.tw.
29 PMO$.tw.
30 eteplirsen.tw.
31 SRP-4045.tw.

32	SRP-4053.tw.
33	AVI-4658.tw.
34	NS-065.tw.
35	NCNP-01.tw.
36	AON.tw.
37	AONs.tw.
38	AO.tw.
39	AOs.tw.
40	ethylene-bridged nucleic acid$.tw.
41	ENA$.tw.
42	DS-5141b.tw.
43	locked nucleic acid$.tw.
44	LNA$.tw.
45	Peptide Nucleic Acids/
46	peptide nucleic acid$.tw.
47	PNA$.tw.
48	tricyclo-DNA$.tw.
49	tcDNA$.tw.
50	Cell-Penetrating Peptides/
51	cell-penetrating peptide$.tw.
52	vivo-morpholino$.tw.
53	or/9-52
54	8 and 53
55	randomized controlled trial.pt.
56	controlled clinical trial.pt.
57	randomized.ab.
58	placebo.ab.
59	drug therapy.fs.
60	randomly.ab.
61	trial.ab.
62	groups.ab.
63	or/55-62
64	54 and 63

65 exp Animals/ not Humans.sh.

66 64 not 65

67 remove duplicates from 66

Appendix 5: Search term for EMBASE

#1	duchenne muscular dystrophy'/de
#2	(duchenne NEAR/5 dystroph*):ab,ti
#3	'muscular dystrophy'/de
#4	'becker muscular dystrophy'/de
#5	(becker NEAR/5 dystroph*):ab,ti
#6	'dystrophinopathy'/de
#7	dystrophinopath*:ab,ti
#8	'dystrophin'/de
#9	xldc:ab,ti OR xldcm:ab,ti OR 'x linked dilated cardiomyopathy':ab,ti
#10	#1 OR #2 OR #3 OR #4 OR #5 OR #6 OR #7 OR #8 OR #9
#11	'antisense oligonucleotide'/exp
#12	(antisense NEAR/1 oligo*):ab,ti OR ('anti sense' NEAR/1 oligo*):ab,ti
#13	'oligonucleotide phosphorothioate'/de
#14	(phospho* NEXT/1 oligonucleotide*):ab,ti
#15	'exon skipping'/de OR (exon* NEAR/5 skip*):ab,ti
#16	2ome*:ab,ti
#17	'2 ome*':ab,ti
#18	'2 o me*':ab,ti
#19	'2o me*':ab,ti
#20	'drisapersen'/de
#21	drisapersen:ab,ti
#22	pro044:ab,ti
#23	bmn044:ab,ti
#24	pro045:ab,ti
#25	bmn045:ab,ti
#26	pro051:ab,ti
#27	bmn051:ab,ti
#28	kyndrisa:ab,ti
#29	pro053:ab,ti
#30	bmn053:ab,ti
#31	'phosphorodiamidate morpholino oligomer*':ab,ti

#32 pmo*:ab,ti

#33 'eteplirsen'/de

#34 eteplirsen:ab,ti

#35 'srp 4045':ab,ti

#36 'srp 4053':ab,ti

#37 'avi 4658':ab,ti

#38 'ns 065':ab,ti

#39 'ncnp-01':ab,ti

#40 aon:ab,ti

#41 aons:ab,ti

#42 ao:ab,ti

#43 aos:ab,ti

#44 'ethylene bridged nucleic acid':ab,ti

#45 enas:ab,ti

#46 'ds 5141b':ab,ti

#47 'locked nucleic acid'/de

#48 'locked nucleic acid*':ab,ti

#49 lna*:ab,ti

#50 'peptide nucleic acid'/de

#51 'peptide nucleic acid*':ab,ti

#52 pna*:ab,ti

#53 'tricyclo dna*':ab,ti

#54 tcdna*:ab,ti

#55 'tc dna*':ab,ti

#56 'cell penetrating peptide'/de

#57 'cell penetrating peptide*':ab,ti

#58 'vivo morpholino*':ab,ti

#59 #11 OR #12 OR #13 OR #14 OR #15 OR #16 OR #17 OR #18 OR #19 OR #20 OR #21 OR #22 OR #23 OR #24 OR #25 OR #26 OR #27 OR #28OR #29 OR #30 OR #31 OR #32 OR #33 OR #34 OR #35 OR #36 OR #37 OR #38 OR #39 OR #40 OR #41 OR #42 OR #43 OR #44 OR #45 OR#46 OR #47 OR #48 OR #49 OR #50 OR #51 OR #52 OR #53 OR #54 OR #55 OR #56 OR #57 OR #58

#60 #10 AND #59

#61 'clinical trial'/de OR 'randomized controlled trial'/de OR 'randomization'/exp

AND random*:ab OR rct*:ab OR trial*:ab OR groups:ab ORplacebo*:ab OR 'drug therapy'/exp

#62	#60 AND #61
#63	#62 NOT ([animals]/lim NOT [humans]/lim)
#64	#1 OR #2 OR #4 OR #5 OR #6 OR #7 OR #8 OR #9
#65	#59 AND #64
#66	#61 AND #65
#67	#66 NOT ([animals]/lim NOT [humans]/lim)
#68	#63 AND [embase]/lim NOT [medline]/lim
#69	#67 AND [embase]/lim NOT [medline]/lim
#70	#68 NOT #69

Appendix 6: Search term for ICHUSHI-web

#1 筋ジストロフィー-Duchenne 型/TH

#2 @筋ジストロフィー/TH

#3 筋ジス/AL and (duchenne/AL or デュシェンヌ/AL or デュシャン/AL)

#4 筋ジス/AL and (becker/AL or ベッカー/AL)

#5 (Dystrophin/TH or ジストロフィン/AL)

#6 ジストロフィノパチー/AL

#7 (X 連鎖/AL or 遺伝/AL) and (心筋症-拡張型/TH or 拡張型心筋症/AL)

#8 #1 or #2 or #3 or #4 or #5 or #6 or #7

#9 "Antisense Oligonucleotides"/TH

#10 (オリゴ/AL and ヌクレオチド/AL) or oligonucleotide/AL or oligodeoxynucleotide/AL or oligodeoxyribonucleotide/AL

#11 (アンチセンス/AL or antisense/AL) and (オリゴ/Al or oligo/AL)

#12 "Phosphorothioate Oligonucleotides"/TH

#13 (ホスホロ/AL or phospho/AL) and ((オリゴ/AL and ヌクレオチド/AL) or oligonucleotide/AL or oligodeoxynucleotide/AL or oligodeoxyribonucleotide/AL)

#14 エクソン/TH

#15 エクソン/AL or エキソン/AL or exon/AL

#16 2'OMe/AL or 2'-OMe/AL or 2'O メチル/AL or 2'O-メチル/AL or 2'-O メチル/AL or 2'-O-メチル/AL

#17 ドリサペルセン/AL or ドリサパーソン/AL or drisapersen/AL

#18 PRO044/AL

#19 BMN044/AL

#20 PRO045/AL

#21 BMN045/AL

#22 PRO051/AL

#23 BMN051/AL

#24 Kyndrisa/AL

#25 PRO053/AL

#26 BMN053/AL

#27 "Phosphorodiamidate Morpholino Oligomer"/AL

#28 PMO/AL

#29 モルフォリノ/AL or モルフォリーノ/AL

#30 エテプリルセン/AL or eteplirsen/AL

#31 SRP-4045/AL

#32 SRP-4053/AL

#33 AVI-4658/AL

#34 NS-065/AL

#35 NCNP-01/AL

#36 AON/AL

#37 AONs/AL

#38 AO/AL

#39 AOs/AL

#40 エチレン架橋核酸/AL or "ethylene bridged nucleic acid"/AL

#41 ENA/AL

#42 DS-5141b/AL

#43 "Locked Nucleic Acid"/TH

#44 (架橋/AL and 核酸/AL) or "locked nucleic acid"/AL

#45 LNA/AL

#46 "Peptide Nucleic Acids"/TH

#47 ペプチド核酸/AL or "peptide nucleic acid"/AL

#48 PNA/AL

#49 tricyclo-DNA/AL or "tricyclo DNA"/AL

#50 tcDNA/AL

#51 "Cell-Penetrating Peptides"/TH

#52 膜透過ペプチド/AL or 細胞透過ペプチド/Al or "cell penetrating peptide"/AL or "cell-penetrating peptide"/AL

#53 #9 or #10 or #11 or #12 or #13 or #14 or #15 or #16 or #17 or #18 or #19 or #20 or #21 or #22 or #23 or #24 or #25 or #26 or #27 or #28 or #29 or #30 or #31 or #32 or #33 or #34 or #35 or #36 or #37 or #38 or #39 or #40 or #41 or #42 or #43 or #44 or #45 or #46 or #47 or #48 or #49 or #50 or #51 or #52

#54 #8 and #53

#55 (#54) and (RD=ランダム化比較試験)

Appendix 7: Search terms for Primary Registries in the WHO Registry Network and in registries approved by the ICMJE

1. DMD AND exon
2. Duchenne AND exon
3. Becker AND exon
4. GSK2402968 AND DMD
5. GSK2402968 AND BMD
6. AVI-4658 AND DMD
7. AVI-4658 AND BMD
8. drisapersen AND DMD
9. drisapersen AND BMD
10. eteplirsen AND DMD
11. eteplirsen AND BMD
12. SRP-4045
13. SRP-4053
14. NS-065/NCNP01
15. PRO044
16. PRO045
17. PRO053

Appendix 8: Adverse events (Cardiac toxicity)

Study or Subgroup	Exon skipping Events	Exon skipping Total	Placebo Events	Placebo Total	Weight	Risk Ratio M-H, Random, 95% CI
**1.5.1 eteplirsen**						
Mendell2013	1	8	0	4	35.7%	1.67 [0.08, 33.75]
**Subtotal (95% CI)**		**8**		**4**	**35.7%**	**1.67 [0.08, 33.75]**
Total events	1		0			
Heterogeneity: Not applicable						
Test for overall effect: Z = 0.33 (P = 0.74)						
**1.5.2 drisapersen**						
Flanigan2014	0	15	0	5		Not estimable
NCT01254019	1	125	0	61	31.8%	1.48 [0.06, 35.72]
NCT01462292	0	35	0	16		Not estimable
Voit2014	1	35	0	18	32.5%	1.58 [0.07, 37.02]
**Subtotal (95% CI)**		**210**		**100**	**64.3%**	**1.53 [0.16, 14.38]**
Total events	2		0			
Heterogeneity: Tau² = 0.00; Chi² = 0.00, df = 1 (P = 0.98); I² = 0%						
Test for overall effect: Z = 0.37 (P = 0.71)						
**Total (95% CI)**		**218**		**104**	**100.0%**	**1.58 [0.26, 9.51]**
Total events	3		0			
Heterogeneity: Tau² = 0.00; Chi² = 0.00, df = 2 (P = 1.00); I² = 0%						
Test for overall effect: Z = 0.50 (P = 0.62)						
Test for subgroup differences: Chi² = 0.00, df = 1 (P = 0.96), I² = 0%						

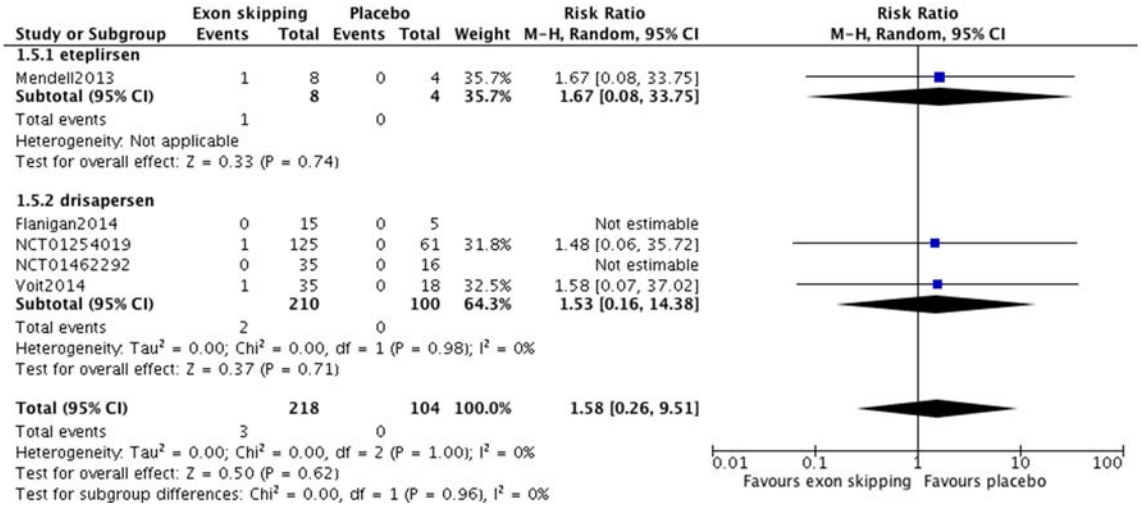

Appendix 9: Adverse events (Gastrointestinal symptoms)

Study or Subgroup	Exon skipping Events	Exon skipping Total	Placebo Events	Placebo Total	Weight	Risk Ratio M-H, Random, 95% CI
**1.9.1 eteplirsen**						
Mendell2013	3	8	2	4	13.3%	0.75 [0.20, 2.83]
**Subtotal (95% CI)**		**8**		**4**	**13.3%**	**0.75 [0.20, 2.83]**
Total events	3		2			
Heterogeneity: Not applicable						
Test for overall effect: Z = 0.42 (P = 0.67)						
**1.9.2 drisapersen**						
Flanigan2014	0	15	0	5		Not estimable
NCT01254019	28	125	13	61	69.3%	1.05 [0.59, 1.88]
NCT01462292	4	35	4	16	15.0%	0.46 [0.13, 1.60]
Voit2014	1	35	0	18	2.4%	1.58 [0.07, 37.02]
**Subtotal (95% CI)**		**210**		**100**	**86.7%**	**0.92 [0.55, 1.55]**
Total events	33		17			
Heterogeneity: Tau² = 0.00; Chi² = 1.51, df = 2 (P = 0.47); I² = 0%						
Test for overall effect: Z = 0.31 (P = 0.76)						
**Total (95% CI)**		**218**		**104**	**100.0%**	**0.90 [0.55, 1.45]**
Total events	36		19			
Heterogeneity: Tau² = 0.00; Chi² = 1.59, df = 3 (P = 0.66); I² = 0%						
Test for overall effect: Z = 0.45 (P = 0.66)						
Test for subgroup differences: Chi² = 0.08, df = 1 (P = 0.78), I² = 0%						

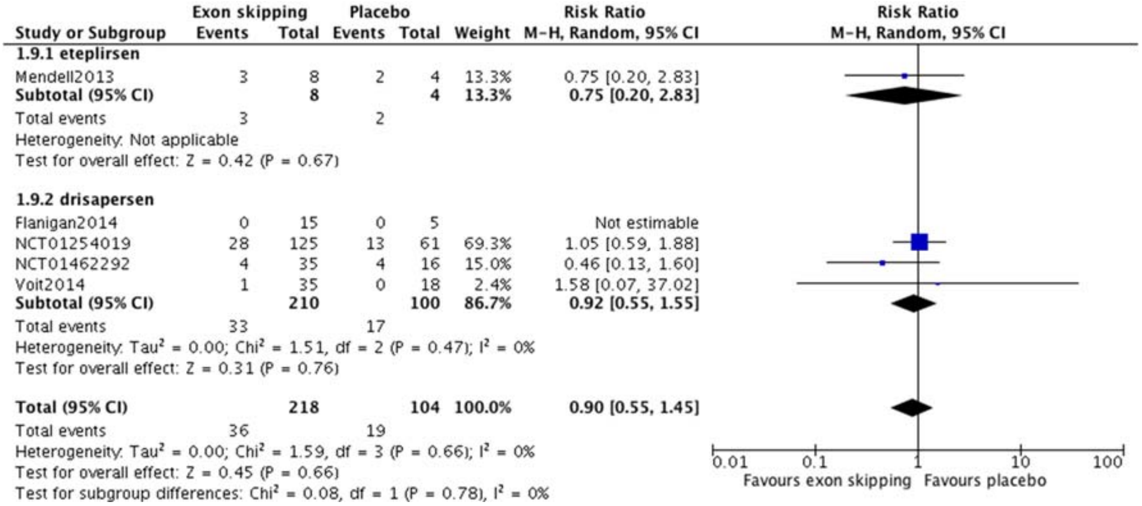

Appendix 10: Adverse events (Hematologic abnormality)

Study or Subgroup	Exon skipping Events	Exon skipping Total	Placebo Events	Placebo Total	Weight	Risk Ratio M-H, Random, 95% CI
**1.13.1 eteplirsen**						
Mendell2013	0	8	0	4		Not estimable
**Subtotal (95% CI)**		**8**		**4**		**Not estimable**
Total events	0		0			
Heterogeneity: Not applicable						
Test for overall effect: Not applicable						
**1.13.2 drisapersen**						
Flanigan2014	0	15	0	5		Not estimable
NCT01254019	1	125	0	61	22.1%	1.48 [0.06, 35.72]
NCT01462292	0	35	0	16		Not estimable
Voit2014	2	35	3	18	77.9%	0.34 [0.06, 1.87]
**Subtotal (95% CI)**		**210**		**100**	**100.0%**	**0.47 [0.11, 2.12]**
Total events	3		3			
Heterogeneity: $Tau^2 = 0.00$; $Chi^2 = 0.64$, df = 1 (P = 0.43); $I^2 = 0\%$						
Test for overall effect: Z = 0.98 (P = 0.33)						
**Total (95% CI)**		**218**		**104**	**100.0%**	**0.47 [0.11, 2.12]**
Total events	3		3			
Heterogeneity: $Tau^2 = 0.00$; $Chi^2 = 0.64$, df = 1 (P = 0.43); $I^2 = 0\%$						
Test for overall effect: Z = 0.98 (P = 0.33)						
Test for subgroup differences: Not applicable						

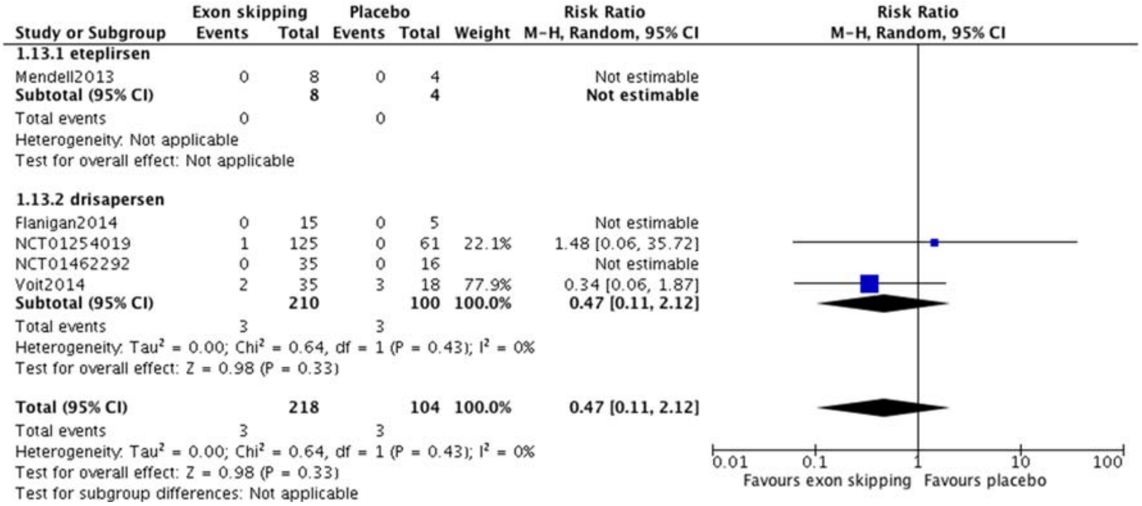

Appendix 11: Adverse events (Hemorrhage)

Study or Subgroup	Exon skipping Events	Exon skipping Total	Placebo Events	Placebo Total	Weight	Risk Ratio M-H, Random, 95% CI
**1.17.1 eteplirsen**						
Mendell2013	2	8	1	4	100.0%	1.00 [0.13, 8.00]
**Subtotal (95% CI)**		**8**		**4**	**100.0%**	**1.00 [0.13, 8.00]**
Total events	2		1			
Heterogeneity: Not applicable						
Test for overall effect: Z = 0.00 (P = 1.00)						
**1.17.2 drisapersen**						
Flanigan2014	0	15	0	5		Not estimable
NCT01254019	0	125	0	61		Not estimable
NCT01462292	0	35	0	16		Not estimable
Voit2014	0	35	0	18		Not estimable
**Subtotal (95% CI)**		**210**		**100**		**Not estimable**
Total events	0		0			
Heterogeneity: Not applicable						
Test for overall effect: Not applicable						
**Total (95% CI)**		**218**		**104**	**100.0%**	**1.00 [0.13, 8.00]**
Total events	2		1			
Heterogeneity: Not applicable						
Test for overall effect: Z = 0.00 (P = 1.00)						
Test for subgroup differences: Not applicable						

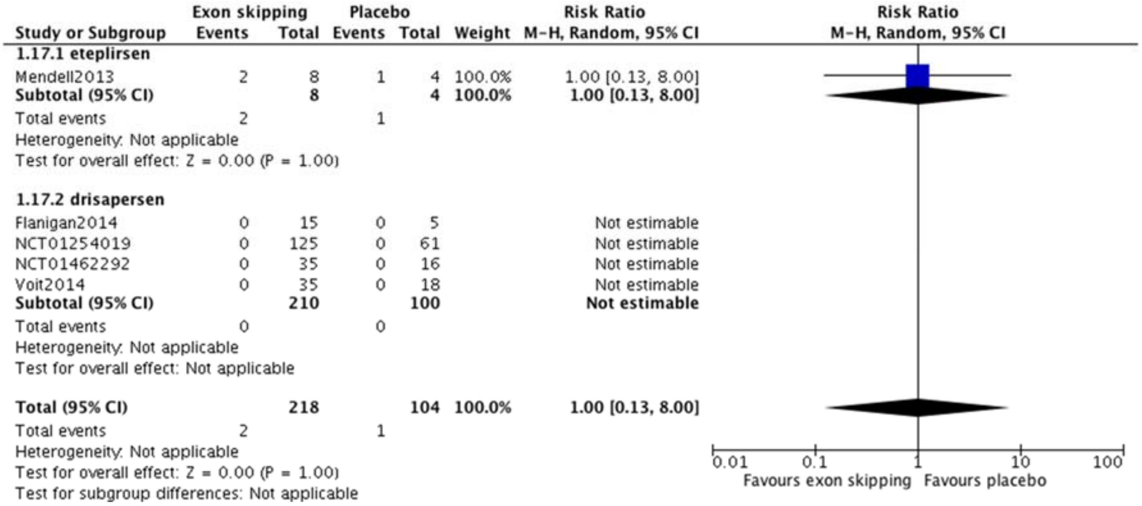

Appendix 12: Adverse events (Hepatic toxicity)

Study or Subgroup	Exon skipping Events	Exon skipping Total	Placebo Events	Placebo Total	Weight	Risk Ratio M-H, Random, 95% CI
**1.21.1 eteplirsen**						
Mendell2013	0	8	0	4		Not estimable
**Subtotal (95% CI)**		**8**		**4**		**Not estimable**
Total events	0		0			
Heterogeneity: Not applicable						
Test for overall effect: Not applicable						
**1.21.2 drisapersen**						
Flanigan2014	0	15	0	5		Not estimable
NCT01254019	1	125	0	61	42.0%	1.48 [0.06, 35.72]
NCT01462292	0	35	0	16		Not estimable
Voit2014	1	35	1	18	58.0%	0.51 [0.03, 7.75]
**Subtotal (95% CI)**		**210**		**100**	**100.0%**	**0.80 [0.10, 6.32]**
Total events	2		1			
Heterogeneity: Tau² = 0.00; Chi² = 0.25, df = 1 (P = 0.62); I² = 0%						
Test for overall effect: Z = 0.21 (P = 0.83)						
**Total (95% CI)**		**218**		**104**	**100.0%**	**0.80 [0.10, 6.32]**
Total events	2		1			
Heterogeneity: Tau² = 0.00; Chi² = 0.25, df = 1 (P = 0.62); I² = 0%						
Test for overall effect: Z = 0.21 (P = 0.83)						
Test for subgroup differences: Not applicable						

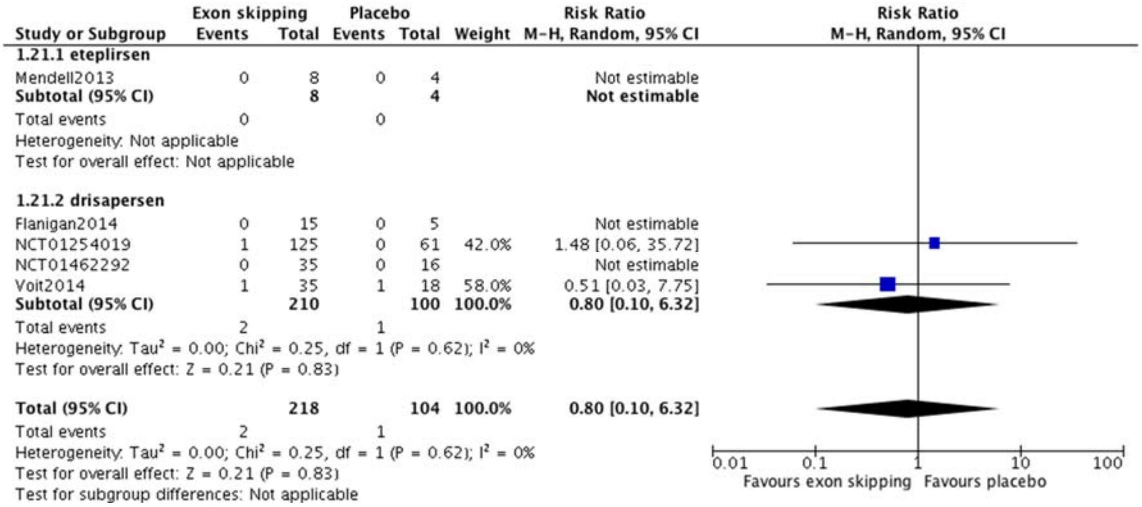

Appendix 13: Adverse events (Inflammation)

Study or Subgroup	Exon skipping Events	Exon skipping Total	Placebo Events	Placebo Total	Weight	Risk Ratio M-H, Random, 95% CI
**1.25.1 eteplirsen**						
Mendell2013	1	8	2	4	6.2%	0.25 [0.03, 2.00]
**Subtotal (95% CI)**		**8**		**4**	**6.2%**	**0.25 [0.03, 2.00]**
Total events	1		2			
Heterogeneity: Not applicable						
Test for overall effect: Z = 1.31 (P = 0.19)						
**1.25.2 drisapersen**						
Flanigan2014	4	15	0	5	3.6%	3.38 [0.21, 53.70]
NCT01254019	38	125	25	61	52.4%	0.74 [0.50, 1.11]
NCT01462292	9	35	2	16	12.2%	2.06 [0.50, 8.45]
Voit2014	13	35	5	18	25.7%	1.34 [0.57, 3.16]
**Subtotal (95% CI)**		**210**		**100**	**93.8%**	**1.04 [0.61, 1.76]**
Total events	64		32			
Heterogeneity: $Tau^2 = 0.09$; $Chi^2 = 4.06$, df = 3 (P = 0.26); $I^2 = 26\%$						
Test for overall effect: Z = 0.13 (P = 0.90)						
**Total (95% CI)**		**218**		**104**	**100.0%**	**0.97 [0.56, 1.66]**
Total events	65		34			
Heterogeneity: $Tau^2 = 0.10$; $Chi^2 = 5.42$, df = 4 (P = 0.25); $I^2 = 26\%$						
Test for overall effect: Z = 0.13 (P = 0.90)						
Test for subgroup differences: $Chi^2 = 1.69$, df = 1 (P = 0.19), $I^2 = 40.7\%$						

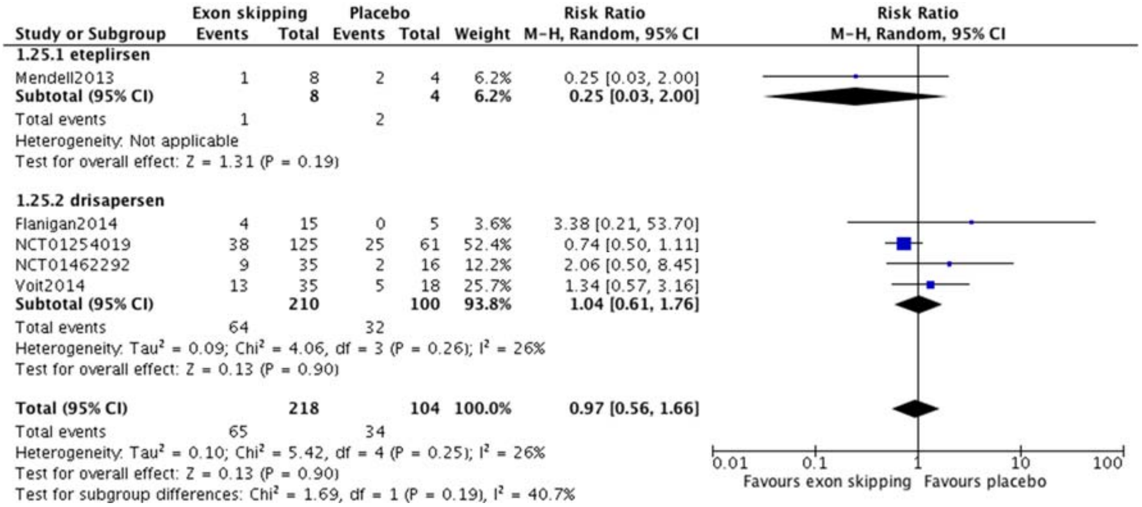

Appendix 14: Adverse events (Pain)

Study or Subgroup	Exon skipping Events	Exon skipping Total	Placebo Events	Placebo Total	Weight	Risk Ratio M-H, Random, 95% CI	Risk Ratio M-H, Random, 95% CI
**1.33.1 eteplirsen**							
Mendell2013	4	8	3	4	25.0%	0.67 [0.27, 1.63]	
**Subtotal (95% CI)**		**8**		**4**	**25.0%**	**0.67 [0.27, 1.63]**	
Total events	4		3				
Heterogeneity: Not applicable							
Test for overall effect: Z = 0.89 (P = 0.37)							
**1.33.2 drisapersen**							
Flanigan2014	0	15	0	5		Not estimable	
NCT01254019	33	125	12	61	58.4%	1.34 [0.75, 2.41]	
NCT01462292	5	35	4	16	14.5%	0.57 [0.18, 1.85]	
Voit2014	1	35	0	18	2.0%	1.58 [0.07, 37.02]	
**Subtotal (95% CI)**		**210**		**100**	**75.0%**	**1.14 [0.68, 1.92]**	
Total events	39		16				
Heterogeneity: Tau² = 0.00; Chi² = 1.67, df = 2 (P = 0.43); I² = 0%							
Test for overall effect: Z = 0.50 (P = 0.61)							
**Total (95% CI)**		**218**		**104**	**100.0%**	**1.00 [0.64, 1.56]**	
Total events	43		19				
Heterogeneity: Tau² = 0.00; Chi² = 2.84, df = 3 (P = 0.42); I² = 0%							
Test for overall effect: Z = 0.01 (P = 0.99)							
Test for subgroup differences: Chi² = 1.04, df = 1 (P = 0.31), I² = 4.2%							

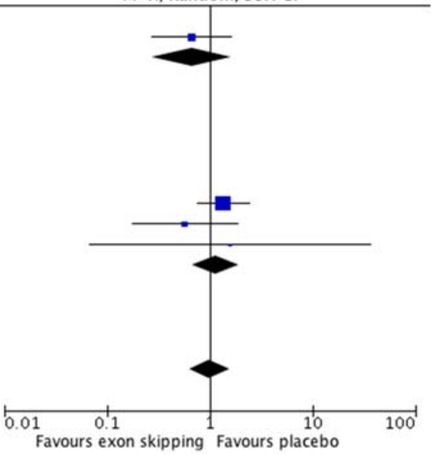

Appendix 15: Adverse events (Others)

Study or Subgroup	Exon skipping Events	Exon skipping Total	Placebo Events	Placebo Total	Weight	Risk Ratio M-H, Random, 95% CI
**1.41.1 eteplirsen**						
Mendell2013	4	8	2	4	16.6%	1.00 [0.30, 3.32]
**Subtotal (95% CI)**		**8**		**4**	**16.6%**	**1.00 [0.30, 3.32]**
Total events	4		2			
Heterogeneity: Not applicable						
Test for overall effect: Z = 0.00 (P = 1.00)						
**1.41.2 drisapersen**						
Flanigan2014	0	15	0	5		Not estimable
NCT01254019	27	125	12	61	64.9%	1.10 [0.60, 2.02]
NCT01462292	6	35	3	16	15.2%	0.91 [0.26, 3.20]
Voit2014	1	35	1	18	3.3%	0.51 [0.03, 7.75]
**Subtotal (95% CI)**		**210**		**100**	**83.4%**	**1.03 [0.60, 1.76]**
Total events	34		16			
Heterogeneity: Tau² = 0.00; Chi² = 0.33, df = 2 (P = 0.85); I² = 0%						
Test for overall effect: Z = 0.11 (P = 0.91)						
**Total (95% CI)**		**218**		**104**	**100.0%**	**1.03 [0.63, 1.67]**
Total events	38		18			
Heterogeneity: Tau² = 0.00; Chi² = 0.33, df = 3 (P = 0.95); I² = 0%						
Test for overall effect: Z = 0.10 (P = 0.92)						
Test for subgroup differences: Chi² = 0.00, df = 1 (P = 0.96), I² = 0%						

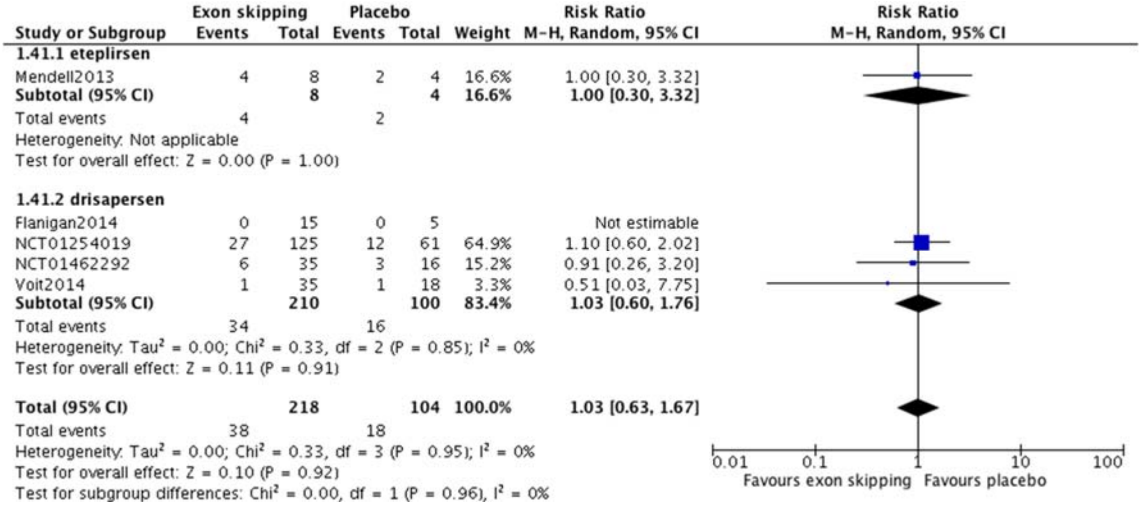

Appendix 16: 6MWT at week 48 (change from baseline, meters)

Study or Subgroup	Exon skipping Mean	Exon skipping SD	Exon skipping Total	Placebo Mean	Placebo SD	Placebo Total	Weight	Mean Difference IV, Random, 95% CI
**1.41.2 Drisapersen**								
NCT01254019	42.32	79.8053	117	52.65	80.0606	59	59.3%	-10.33 [-35.36, 14.70]
Voit2014	-7.2	53.104	33	24.7	52.8	17	40.7%	-31.90 [-62.86, -0.94]
**Subtotal (95% CI)**			**150**			**76**	**100.0%**	**-19.11 [-39.88, 1.66]**
Heterogeneity: Tau² = 26.37; Chi² = 1.13, df = 1 (P = 0.29); I² = 11%								
Test for overall effect: Z = 1.80 (P = 0.07)								
**Total (95% CI)**			**150**			**76**	**100.0%**	**-19.11 [-39.88, 1.66]**
Heterogeneity: Tau² = 26.37; Chi² = 1.13, df = 1 (P = 0.29); I² = 11%								
Test for overall effect: Z = 1.80 (P = 0.07)								
Test for subgroup differences: Not applicable								

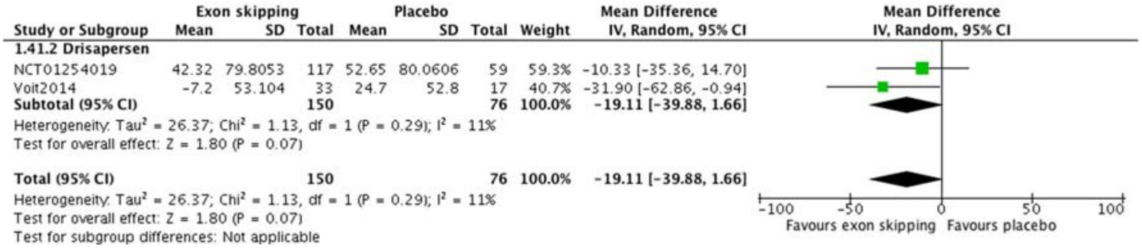

For mean difference, gain of distance is shown in negative numbers and loss of distance is shown in positive numbers

Appendix 17: Timed test at week 24, time taken for 4 stairs climb, (change from baseline, seconds)

Study or Subgroup	Exon skipping Mean	Exon skipping SD	Exon skipping Total	Placebo Mean	Placebo SD	Placebo Total	Weight	Mean Difference IV, Random, 95% CI
**1.45.1 Eteplirsen**								
Mendell2013	4.85	9.788	8	-1.22	1.597	4	1.3%	6.07 [-0.89, 13.03]
**Subtotal (95% CI)**			**8**			**4**	**1.3%**	**6.07 [-0.89, 13.03]**
Heterogeneity: Not applicable								
Test for overall effect: Z = 1.71 (P = 0.09)								
**1.45.2 Drisapersen**								
NCT01462292	0.1734	1.2346	35	0.59	1.232	16	48.6%	-0.42 [-1.15, 0.31]
Voit2014	-0.0967	1.2346	35	0	1.232	18	50.1%	-0.10 [-0.80, 0.60]
**Subtotal (95% CI)**			**70**			**34**	**98.7%**	**-0.25 [-0.76, 0.25]**
Heterogeneity: $Tau^2 = 0.00$; $Chi^2 = 0.38$, df = 1 (P = 0.54); $I^2 = 0\%$								
Test for overall effect: Z = 0.97 (P = 0.33)								
**Total (95% CI)**			**78**			**38**	**100.0%**	**-0.17 [-0.97, 0.62]**
Heterogeneity: $Tau^2 = 0.20$; $Chi^2 = 3.53$, df = 2 (P = 0.17); $I^2 = 43\%$								
Test for overall effect: Z = 0.43 (P = 0.67)								
Test for subgroup differences: $Chi^2 = 3.15$, df = 1 (P = 0.08), $I^2 = 68.3\%$								

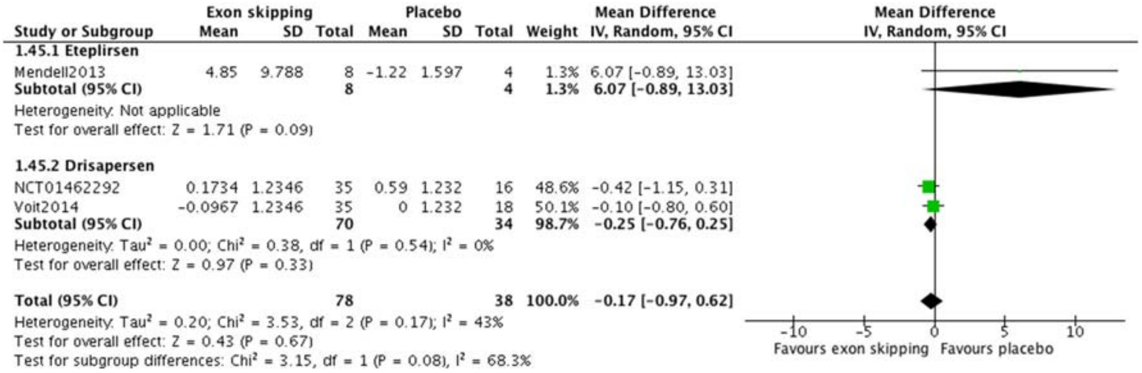

Standard deviations (SDs) were not provided for Voit 2014, therefore were substituted by SDs reported in NCT01462292.

Appendix 18: Timed test at week 24, time taken for 4 stairs descent, (change from baseline, seconds)

Study or Subgroup	Exon skipping Mean	SD	Total	Placebo Mean	SD	Total	Weight	Mean Difference IV, Random, 95% CI
**1.49.2 Drisapersen**								
NCT01462292	0.054	1.8454	35	0.6	1.892	16	48.0%	-0.55 [-1.66, 0.56]
Voit2014	-0.259	1.8454	35	0	1.892	18	52.0%	-0.26 [-1.33, 0.81]
**Subtotal (95% CI)**			**70**			**34**	**100.0%**	**-0.40 [-1.17, 0.37]**
Heterogeneity: $Tau^2 = 0.00$; $Chi^2 = 0.13$, df = 1 (P = 0.71); $I^2 = 0\%$								
Test for overall effect: Z = 1.01 (P = 0.31)								
**Total (95% CI)**			**70**			**34**	**100.0%**	**-0.40 [-1.17, 0.37]**
Heterogeneity: $Tau^2 = 0.00$; $Chi^2 = 0.13$, df = 1 (P = 0.71); $I^2 = 0\%$								
Test for overall effect: Z = 1.01 (P = 0.31)								
Test for subgroup differences: Not applicable								

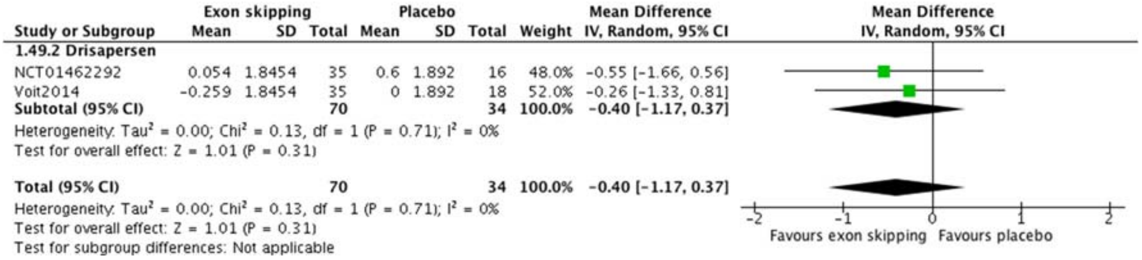

Standard deviations (SDs) were not provided for Voit 2014, therefore were substituted by SDs reported in NCT01462292.

Appendix 19: Timed test at week 24, time taken for 10 minutes walk/run, (change from baseline, seconds)

	Exon skipping			Placebo				Mean Difference
Study or Subgroup	Mean	SD	Total	Mean	SD	Total	Weight	IV, Random, 95% CI
**1.53.1 Eteplirsen**								
Mendell2013	2.81	7.357	8	-0.65	0.985	4	1.8%	3.46 [-1.73, 8.65]
**Subtotal (95% CI)**			**8**			**4**	**1.8%**	**3.46 [-1.73, 8.65]**
Heterogeneity: Not applicable								
Test for overall effect: Z = 1.31 (P = 0.19)								
**1.53.2 Drisapersen**								
NCT01462292	0.2474	0.7782	35	-0.04	0.784	16	48.7%	0.29 [-0.18, 0.75]
Voit2014	-0.384	0.7782	35	0	0.784	18	49.5%	-0.38 [-0.83, 0.06]
**Subtotal (95% CI)**			**70**			**34**	**98.2%**	**-0.05 [-0.71, 0.61]**
Heterogeneity: $Tau^2 = 0.17$; $Chi^2 = 4.21$, df = 1 (P = 0.04); $I^2 = 76\%$								
Test for overall effect: Z = 0.15 (P = 0.88)								
**Total (95% CI)**			**78**			**38**	**100.0%**	**0.01 [-0.69, 0.72]**
Heterogeneity: $Tau^2 = 0.21$; $Chi^2 = 5.97$, df = 2 (P = 0.05); $I^2 = 66\%$								
Test for overall effect: Z = 0.03 (P = 0.97)								
Test for subgroup differences: $Chi^2 = 1.73$, df = 1 (P = 0.19), $I^2 = 42.2\%$								

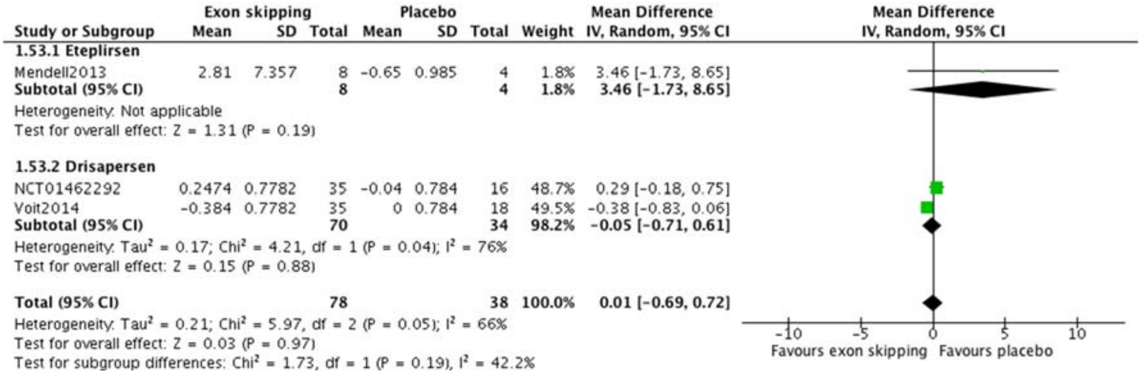

Standard deviations (SDs) were not provided for Voit 2014, therefore were substituted by SDs reported in NCT01462292.

Appendix 20: Timed test at week 24, time taken to rise from floor, (change from baseline, seconds)

	Exon skipping			Placebo				Mean Difference
Study or Subgroup	Mean	SD	Total	Mean	SD	Total	Weight	IV, Random, 95% CI
**1.57.1 Eteplirsen**								
Mendell2013	5.125	10.262	8	-0.7	1.14	4	3.2%	5.83 [-1.37, 13.02]
**Subtotal (95% CI)**			**8**			**4**	**3.2%**	**5.83 [-1.37, 13.02]**
Heterogeneity: Not applicable								
Test for overall effect: Z = 1.59 (P = 0.11)								
**1.57.2 Drisapersen**								
NCT01462292	1.7314	2.7573	35	1.12	2.8	16	46.9%	0.61 [-1.04, 2.26]
Voit2014	0.109	2.7573	35	0	2.8	18	49.9%	0.11 [-1.47, 1.69]
**Subtotal (95% CI)**			**70**			**34**	**96.8%**	**0.35 [-0.79, 1.49]**
Heterogeneity: Tau^2 = 0.00; Chi^2 = 0.19, df = 1 (P = 0.67); I^2 = 0%								
Test for overall effect: Z = 0.60 (P = 0.55)								
**Total (95% CI)**			**78**			**38**	**100.0%**	**0.53 [-0.77, 1.82]**
Heterogeneity: Tau^2 = 0.22; Chi^2 = 2.35, df = 2 (P = 0.31); I^2 = 15%								
Test for overall effect: Z = 0.80 (P = 0.43)								
Test for subgroup differences: Chi^2 = 2.17, df = 1 (P = 0.14), I^2 = 53.9%								

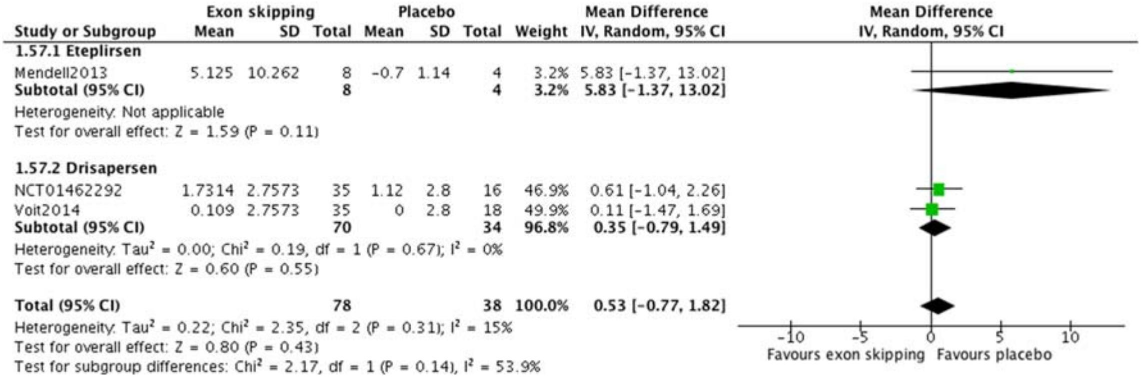

Standard deviations (SDs) were not provided for Voit 2014, therefore were substituted by SDs reported in NCT01462292.

Appendix 21: QOL (PedsQL) at week 48, parents, (change from baseline)

Study or Subgroup	Exon skipping Mean	Exon skipping SD	Exon skipping Total	Placebo Mean	Placebo SD	Placebo Total	Weight	Mean Difference IV, Random, 95% CI
**1.61.2 Drisapersen**								
NCT01254019	1.19	11.269	117	0.11	11.064	58	68.1%	1.08 [-2.42, 4.58]
Voit2014	0.8659	7.1599	32	3.98	11.96	18	31.9%	-3.11 [-9.17, 2.94]
**Subtotal (95% CI)**			**149**			**76**	**100.0%**	**-0.26 [-4.09, 3.57]**
Heterogeneity: $Tau^2 = 2.42$; $Chi^2 = 1.38$, df = 1 (P = 0.24); $I^2 = 28\%$								
Test for overall effect: Z = 0.13 (P = 0.89)								
**Total (95% CI)**			**149**			**76**	**100.0%**	**-0.26 [-4.09, 3.57]**
Heterogeneity: $Tau^2 = 2.42$; $Chi^2 = 1.38$, df = 1 (P = 0.24); $I^2 = 28\%$								
Test for overall effect: Z = 0.13 (P = 0.89)								
Test for subgroup differences: Not applicable								

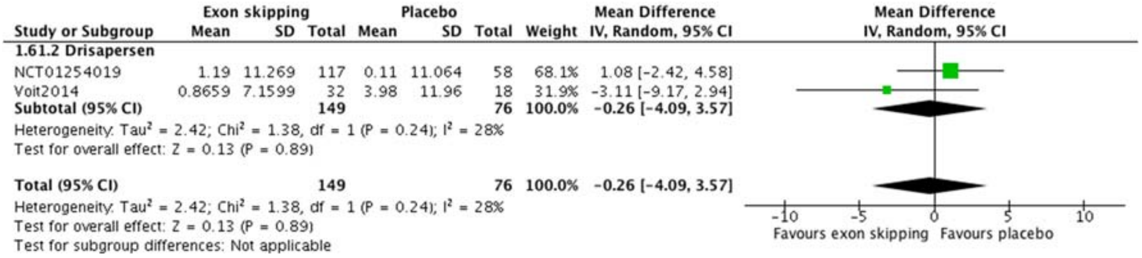

For mean difference, gain of score is shown in negative numbers and loss of score is shown in positive numbers

Appendix 22: QOL (PedsQL) at week 48, patient, (change from baseline)

Study or Subgroup	Exon skipping Mean	SD	Total	Placebo Mean	SD	Total	Weight	Mean Difference IV, Random, 95% CI
**1.63.2 Drisapersen**								
NCT01254019	-1.36	11.36	114	-0.52	11.313	57	76.8%	-0.84 [-4.44, 2.76]
Voit2014	-4.179	8.5009	31	-0.37	12.25	17	23.2%	-3.81 [-10.36, 2.74]
**Subtotal (95% CI)**			**145**			**74**	**100.0%**	**-1.53 [-4.69, 1.63]**
Heterogeneity: $Tau^2 = 0.00$; $Chi^2 = 0.61$, df = 1 (P = 0.44); $I^2 = 0\%$								
Test for overall effect: Z = 0.95 (P = 0.34)								
**Total (95% CI)**			**145**			**74**	**100.0%**	**-1.53 [-4.69, 1.63]**
Heterogeneity: $Tau^2 = 0.00$; $Chi^2 = 0.61$, df = 1 (P = 0.44); $I^2 = 0\%$								
Test for overall effect: Z = 0.95 (P = 0.34)								
Test for subgroup differences: Not applicable								

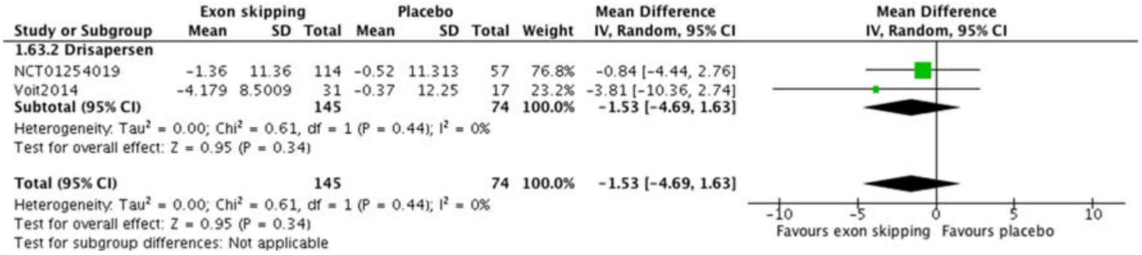

For mean difference, gain of score is shown in negative numbers and loss of score is shown in positive numbers

Appendix 23: Number of participants who withdrew from the study

Study or Subgroup	Exon skipping Events	Exon skipping Total	Placebo Events	Placebo Total	Weight	Risk Ratio M-H, Random, 95% CI
**1.67.1 eteplirsen**						
Mendell2013	0	8	0	4		Not estimable
**Subtotal (95% CI)**		**8**		**4**		**Not estimable**
Total events	0		0			
Heterogeneity: Not applicable						
Test for overall effect: Not applicable						
**1.67.2 drisapersen**						
Flanigan2014	0	15	0	5		Not estimable
NCT01254019	4	125	1	61	100.0%	1.95 [0.22, 17.09]
NCT01462292	0	35	0	16		Not estimable
Voit2014	0	35	0	18		Not estimable
**Subtotal (95% CI)**		**210**		**100**	**100.0%**	**1.95 [0.22, 17.09]**
Total events	4		1			
Heterogeneity: Not applicable						
Test for overall effect: Z = 0.60 (P = 0.55)						
**Total (95% CI)**		**218**		**104**	**100.0%**	**1.95 [0.22, 17.09]**
Total events	4		1			
Heterogeneity: Not applicable						
Test for overall effect: Z = 0.60 (P = 0.55)						
Test for subgroup differences: Not applicable						

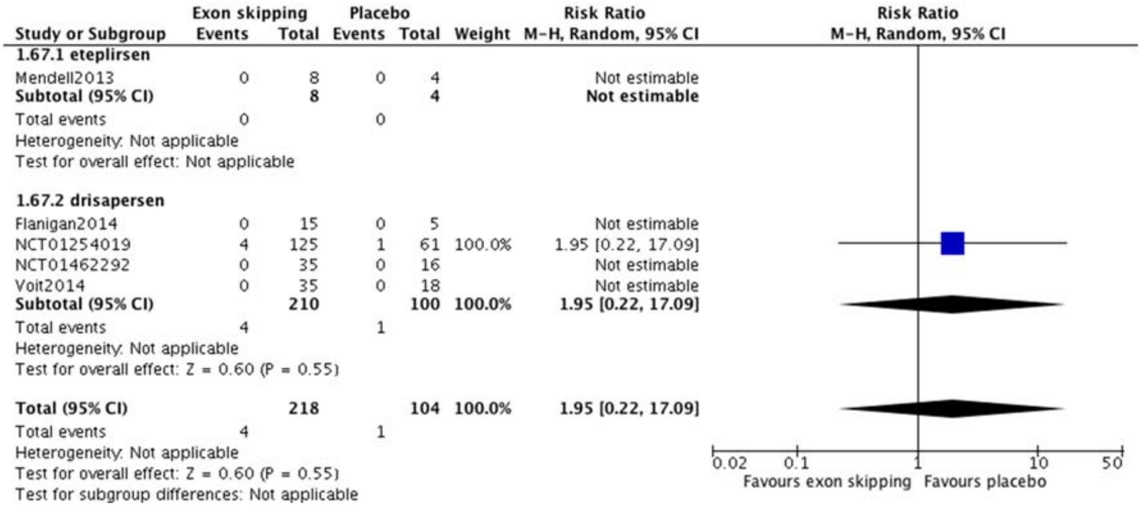

Appendix 24: 6MWT at week 24 (change from baseline, meters), eteplirsen vs placebo

	Exon skipping			Placebo				Mean Difference
Study or Subgroup	Mean	SD	Total	Mean	SD	Total	Weight	IV, Random, 95% CI
**1.93.1 Eteplirsen**								
Mendell2013	64.25	62.801	8	25.8	61.2	4	100.0%	38.45 [−35.65, 112.55]
**Subtotal (95% CI)**			**8**			**4**	**100.0%**	**38.45 [−35.65, 112.55]**
Heterogeneity: Not applicable								
Test for overall effect: Z = 1.02 (P = 0.31)								
**Total (95% CI)**			**8**			**4**	**100.0%**	**38.45 [−35.65, 112.55]**
Heterogeneity: Not applicable								
Test for overall effect: Z = 1.02 (P = 0.31)								
Test for subgroup differences: Not applicable								

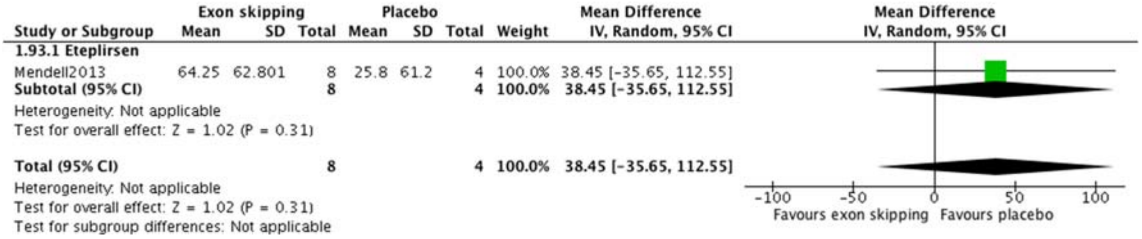

For mean difference, gain of distance is shown in negative numbers and loss of distance is shown in positive numbers

Appendix 25: 6MWT at week 24 (change from baseline, meters), drisapersen vs placebo

Study or Subgroup	Exon skipping Mean	SD	Total	Placebo Mean	SD	Total	Weight	Mean Difference IV, Random, 95% CI
**1.37.1 Drisapersen**								
NCT01254019	24.34	64.284	122	29.11	63.5231	59	42.8%	-4.77 [-24.59, 15.05]
NCT01462292	1.39	41.6495	35	10.98	42.664	16	26.8%	-9.59 [-34.64, 15.46]
Voit2014	-16.21	39.535	31	3.6	38.8	16	30.3%	-19.81 [-43.37, 3.75]
**Subtotal (95% CI)**			**188**			**91**	**100.0%**	**-10.62 [-23.60, 2.35]**
Heterogeneity: Tau² = 0.00; Chi² = 0.93, df = 2 (P = 0.63); I² = 0%								
Test for overall effect: Z = 1.60 (P = 0.11)								
**Total (95% CI)**			**188**			**91**	**100.0%**	**-10.62 [-23.60, 2.35]**
Heterogeneity: Tau² = 0.00; Chi² = 0.93, df = 2 (P = 0.63); I² = 0%								
Test for overall effect: Z = 1.60 (P = 0.11)								
Test for subgroup differences: Not applicable								

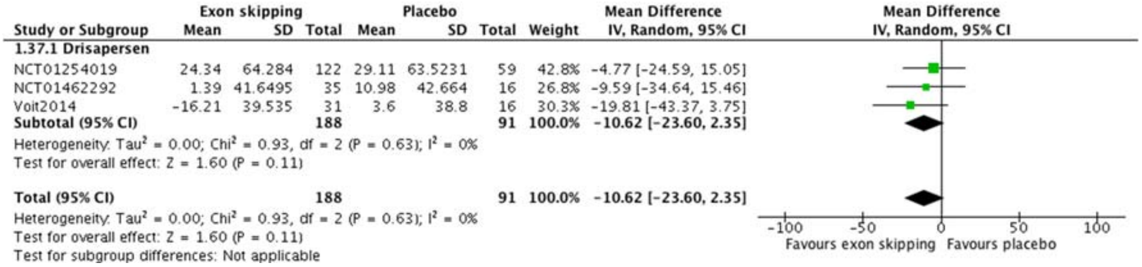

For mean difference, gain of distance is shown in negative numbers and loss of distance is shown in positive numbers

Appendix 26: NSAA at week 24 (change from baseline), eteplirsen vs placebo

Study or Subgroup	Exon skipping Mean	Exon skipping SD	Exon skipping Total	Placebo Mean	Placebo SD	Placebo Total	Weight	Mean Difference IV, Random, 95% CI
**1.93.1 Eteplirsen**								
Mendell2013	4.15	6.438	8	-3.3	2.5	4	100.0%	7.45 [2.36, 12.54]
**Subtotal (95% CI)**			**8**			**4**	**100.0%**	**7.45 [2.36, 12.54]**
Heterogeneity: Not applicable								
Test for overall effect: Z = 2.87 (P = 0.004)								
**Total (95% CI)**			**8**			**4**	**100.0%**	**7.45 [2.36, 12.54]**
Heterogeneity: Not applicable								
Test for overall effect: Z = 2.87 (P = 0.004)								
Test for subgroup differences: Not applicable								

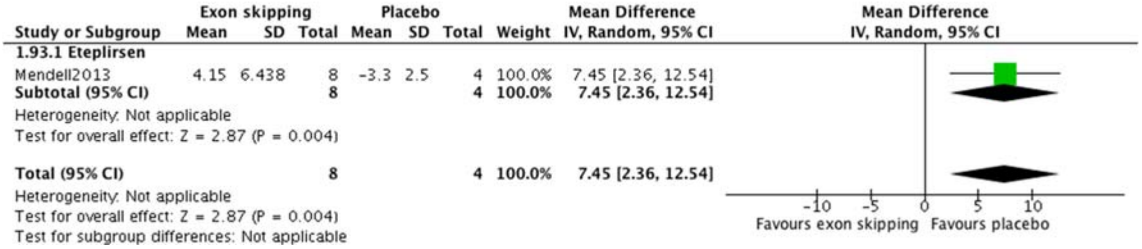

For mean difference, gain of score is shown in negative numbers and loss of score is shown in positive numbers

Appendix 27: NSAA at week 24 (change from baseline), drisapersen vs placebo

	Exon skipping			Placebo				Mean Difference
Study or Subgroup	Mean	SD	Total	Mean	SD	Total	Weight	IV, Random, 95% CI
**1.2.2 Drisapersen**								
NCT01462292	0.946	3.326	35	0.6	3.36	16	49.0%	0.35 [−1.64, 2.33]
Voit2014	−1.67	3.326	35	0	3.36	18	51.0%	−1.67 [−3.57, 0.23]
**Subtotal (95% CI)**			**70**			**34**	**100.0%**	**−0.68 [−2.66, 1.29]**
Heterogeneity: $Tau^2 = 1.05$; $Chi^2 = 2.07$, df = 1 (P = 0.15); $I^2 = 52\%$								
Test for overall effect: Z = 0.68 (P = 0.50)								
**Total (95% CI)**			**70**			**34**	**100.0%**	**−0.68 [−2.66, 1.29]**
Heterogeneity: $Tau^2 = 1.05$; $Chi^2 = 2.07$, df = 1 (P = 0.15); $I^2 = 52\%$								
Test for overall effect: Z = 0.68 (P = 0.50)								
Test for subgroup differences: Not applicable								

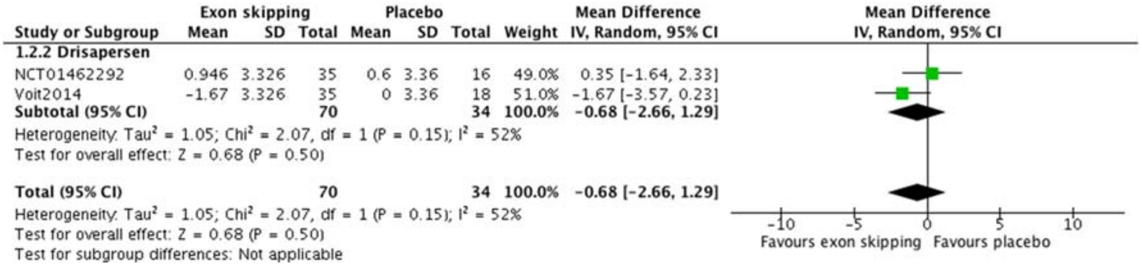

For mean difference, gain of score is shown in negative numbers and loss of score is shown in positive numbers

Appendix 28: 6MWT at week 24, (change from baseline, meters), drisapersen 6mg/kg/time weekly injection

	Exon skipping			Placebo				Mean Difference
Study or Subgroup	Mean	SD	Total	Mean	SD	Total	Weight	IV, Random, 95% CI
**1.1.2 Drisapersen**								
NCT01254019	24.34	64.284	122	29.11	63.523	59	41.5%	-4.77 [-24.59, 15.05]
NCT01462292	-16.12	42.176	18	10.98	42.664	16	28.3%	-27.10 [-55.68, 1.48]
Voit2014	-31.5	39.2	16	3.6	38.8	16	30.2%	-35.10 [-62.13, -8.07]
**Subtotal (95% CI)**			**156**			**91**	**100.0%**	**-20.24 [-39.59, -0.89]**
Heterogeneity: Tau^2 = 132.38; Chi^2 = 3.64, df = 2 (P = 0.16); I^2 = 45%								
Test for overall effect: Z = 2.05 (P = 0.04)								
**Total (95% CI)**			**156**			**91**	**100.0%**	**-20.24 [-39.59, -0.89]**
Heterogeneity: Tau^2 = 132.38; Chi^2 = 3.64, df = 2 (P = 0.16); I^2 = 45%								
Test for overall effect: Z = 2.05 (P = 0.04)								
Test for subgroup differences: Not applicable								

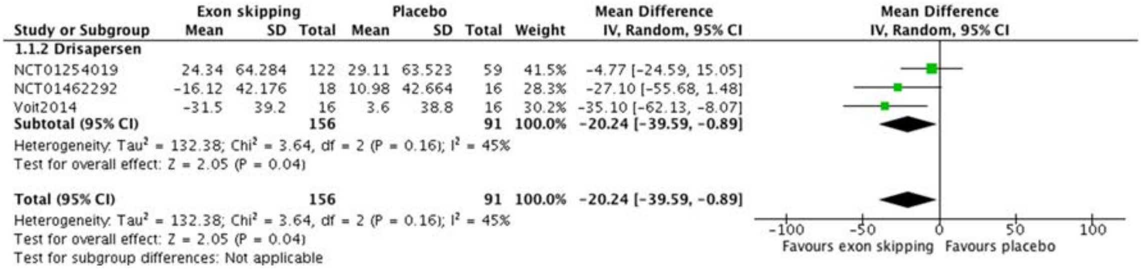

For mean difference, gain of distance is shown in negative numbers and loss of distance is shown in positive numbers

Appendix 29: Adverse events (Injection site reaction, drisapersen 6mg/kg/time weekly injection)

Study or Subgroup	Exon skipping Events	Exon skipping Total	Placebo Events	Placebo Total	Weight	Risk Ratio M-H, Random, 95% CI
**1.5.2 drisapersen**						
Flanigan2014	5	6	1	5	11.8%	4.17 [0.70, 24.94]
NCT01254019	62	125	4	61	31.7%	7.56 [2.89, 19.83]
NCT01462292	9	18	3	16	25.5%	2.67 [0.87, 8.17]
Voit2014	9	18	4	18	30.9%	2.25 [0.84, 5.99]
**Subtotal (95% CI)**		**167**		**100**	**100.0%**	**3.71 [1.93, 7.15]**
Total events	85		12			
Heterogeneity: Tau² = 0.11; Chi² = 3.98, df = 3 (P = 0.26); I² = 25%						
Test for overall effect: Z = 3.92 (P < 0.0001)						
**Total (95% CI)**		**167**		**100**	**100.0%**	**3.71 [1.93, 7.15]**
Total events	85		12			
Heterogeneity: Tau² = 0.11; Chi² = 3.98, df = 3 (P = 0.26); I² = 25%						
Test for overall effect: Z = 3.92 (P < 0.0001)						
Test for subgroup differences: Not applicable						

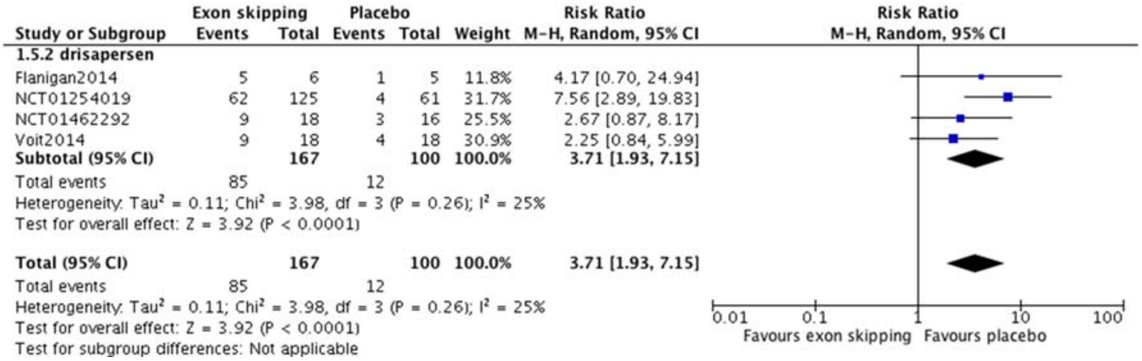

Appendix 30: Adverse events (Renal toxicity, drisapersen 6mg/kg/time weekly injection)

Study or Subgroup	Exon skipping Events	Exon skipping Total	Placebo Events	Placebo Total	Weight	Risk Ratio M-H, Random, 95% CI
**1.33.2 drisapersen**						
Flanigan2014	0	6	0	5		Not estimable
NCT01254019	42	125	11	61	73.6%	1.86 [1.03, 3.36]
NCT01462292	3	18	2	16	9.3%	1.33 [0.25, 7.00]
Voit2014	6	18	3	18	17.1%	2.00 [0.59, 6.79]
**Subtotal (95% CI)**		**167**		**100**	**100.0%**	**1.83 [1.10, 3.03]**
Total events	51		16			
Heterogeneity: $Tau^2 = 0.00$; $Chi^2 = 0.16$, df = 2 (P = 0.92); $I^2 = 0\%$						
Test for overall effect: Z = 2.34 (P = 0.02)						
**Total (95% CI)**		**167**		**100**	**100.0%**	**1.83 [1.10, 3.03]**
Total events	51		16			
Heterogeneity: $Tau^2 = 0.00$; $Chi^2 = 0.16$, df = 2 (P = 0.92); $I^2 = 0\%$						
Test for overall effect: Z = 2.34 (P = 0.02)						
Test for subgroup differences: Not applicable						

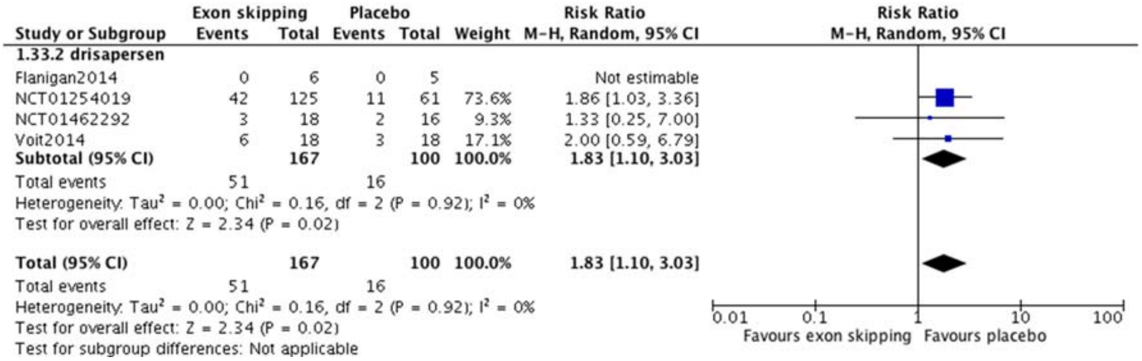

Appendix 31: 6MWT at week 24 (change from baseline, meters), fixed-effect meta-analysis

Study or Subgroup	Exon skipping Mean	Exon skipping SD	Exon skipping Total	Placebo Mean	Placebo SD	Placebo Total	Weight	Mean Difference IV, Fixed, 95% CI
**1.1.1 Eteplirsen**								
Mendell2013	64.25	62.801	8	25.8	61.2	4	3.0%	38.45 [-35.65, 112.55]
**Subtotal (95% CI)**			**8**			**4**	**3.0%**	**38.45 [-35.65, 112.55]**
Heterogeneity: Not applicable								
Test for overall effect: Z = 1.02 (P = 0.31)								
**1.1.2 Drisapersen**								
NCT01254019	24.34	64.284	122	29.11	63.523	59	41.6%	-4.77 [-24.59, 15.05]
NCT01462292	1.39	41.6495	35	10.98	42.664	16	26.0%	-9.59 [-34.64, 15.46]
Voit2014	-16.21	39.535	31	3.6	38.8	16	29.4%	-19.81 [-43.37, 3.75]
**Subtotal (95% CI)**			**188**			**91**	**97.0%**	**-10.62 [-23.60, 2.35]**
Heterogeneity: Chi² = 0.93, df = 2 (P = 0.63); I² = 0%								
Test for overall effect: Z = 1.60 (P = 0.11)								
**Total (95% CI)**			**196**			**95**	**100.0%**	**-9.16 [-21.94, 3.62]**
Heterogeneity: Chi² = 2.56, df = 3 (P = 0.46); I² = 0%								
Test for overall effect: Z = 1.41 (P = 0.16)								
Test for subgroup differences: Chi² = 1.63, df = 1 (P = 0.20), I² = 38.8%								

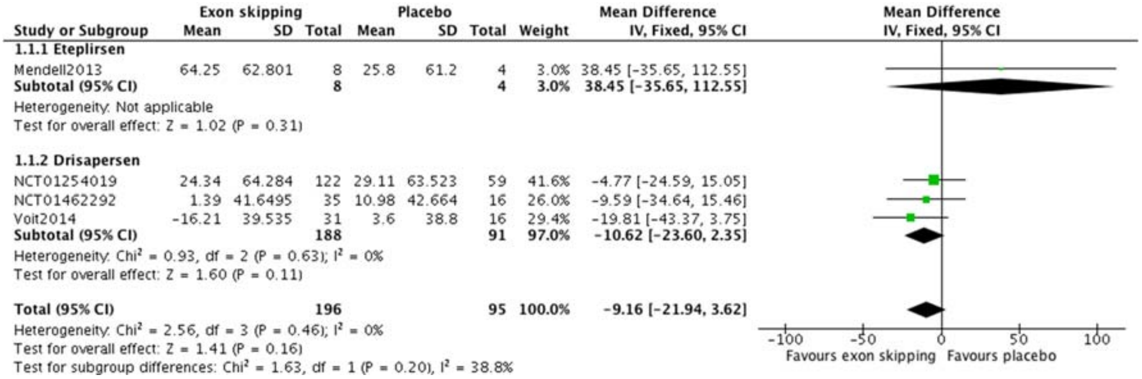

For mean difference, gain of distance is shown in negative numbers and loss of distance is shown in positive numbers

Appendix 32: NSAA at week 24 (change from baseline), fixed-effect meta-analysis

	Exon skipping			Placebo				Mean Difference
**Study or Subgroup**	**Mean**	**SD**	**Total**	**Mean**	**SD**	**Total**	**Weight**	**IV, Fixed, 95% CI**
**1.3.1 Eteplirsen**								
Mendell2013	4.15	6.438	8	-3.3	2.5	4	6.8%	7.45 [2.36, 12.54]
**Subtotal (95% CI)**			**8**			**4**	**6.8%**	**7.45 [2.36, 12.54]**
Heterogeneity: Not applicable								
Test for overall effect: Z = 2.87 (P = 0.004)								
**1.3.2 Drisapersen**								
NCT01462292	0.946	3.326	35	0.6	3.36	16	44.8%	0.35 [-1.64, 2.33]
Voit2014	-1.67	3.326	35	0	3.36	18	48.5%	-1.67 [-3.57, 0.23]
**Subtotal (95% CI)**			**70**			**34**	**93.2%**	**-0.70 [-2.07, 0.67]**
Heterogeneity: Chi^2 = 2.07, df = 1 (P = 0.15); I^2 = 52%								
Test for overall effect: Z = 1.00 (P = 0.32)								
**Total (95% CI)**			**78**			**38**	**100.0%**	**-0.15 [-1.47, 1.18]**
Heterogeneity: Chi^2 = 11.26, df = 2 (P = 0.004); I^2 = 82%								
Test for overall effect: Z = 0.22 (P = 0.83)								
Test for subgroup differences: Chi^2 = 9.19, df = 1 (P = 0.002), I^2 = 89.1%								

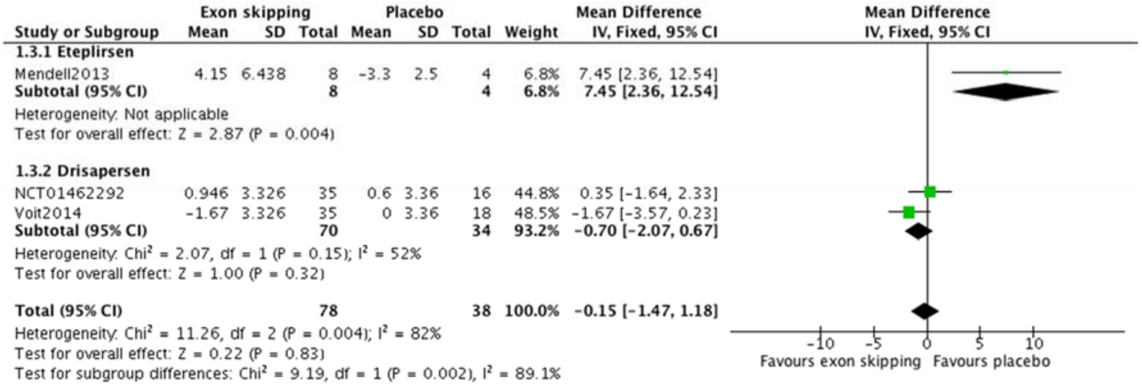

For mean difference, gain of score is shown in negative numbers and loss of score is shown in positive numbers

Appendix 33: 6MWT at week 24 (change from baseline, meters), excluding Mendell 2013 and Voit 2014 which were considered to possess high risk of bias

	Exon skipping			Placebo				Mean Difference
Study or Subgroup	Mean	SD	Total	Mean	SD	Total	Weight	IV, Random, 95% CI
**1.1.2 Drisapersen**								
NCT01254019	24.34	64.284	122	29.11	63.5231	59	61.5%	-4.77 [-24.59, 15.05]
NCT01462292	1.39	41.6495	35	10.98	42.664	16	38.5%	-9.59 [-34.64, 15.46]
**Subtotal (95% CI)**			**157**			**75**	**100.0%**	**-6.63 [-22.17, 8.92]**
Heterogeneity: $Tau^2 = 0.00; Chi^2 = 0.09, df = 1 (P = 0.77); I^2 = 0\%$								
Test for overall effect: Z = 0.84 (P = 0.40)								
**Total (95% CI)**			**157**			**75**	**100.0%**	**-6.63 [-22.17, 8.92]**
Heterogeneity: $Tau^2 = 0.00; Chi^2 = 0.09, df = 1 (P = 0.77); I^2 = 0\%$								
Test for overall effect: Z = 0.84 (P = 0.40)								
Test for subgroup differences: Not applicable								

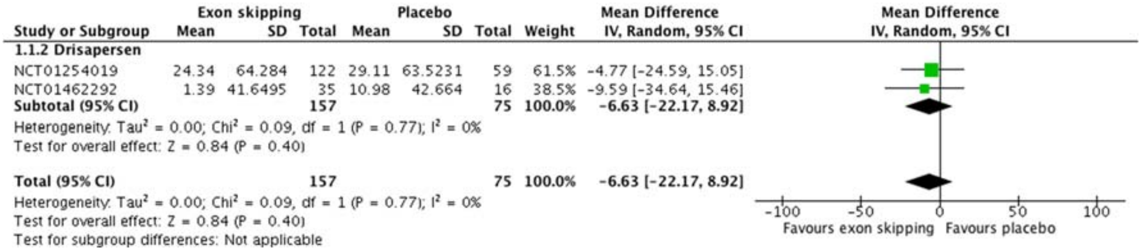

For mean difference, gain of distance is shown in negative numbers and loss of distance is shown in positive numbers

Appendix table 1: Characteristics of excluded studies

Study ID	Reason for exclusion
**Nakamura 2011[1]**	Not double-blind randomized-controlled trial
**Goemans 2011[2]**	Not double-blind randomized-controlled trial
**Gerralda 2011[3]**	Not double-blind randomized-controlled trial
**Cirak 2012[4]**	Not double-blind randomized-controlled trial
**Rodino-Kaplac 2013[5]**	Not double-blind randomized-controlled trial
**NCT01128855**	Secondary publication or studies of included studies[a]
**NCT01153932**	Secondary publication or studies of included studies[b]
**EUCTR2010-018412-32-Outside-EU/EEA**	Secondary publication or studies of included studies[b]
**EUCTR2010-018412-32-NL**	Secondary publication or studies of included studies[b]
**EUCTR2010-018412-32-GB**	Secondary publication or studies of included studies[b]
**EUCTR2010-018412-32-FR**	Secondary publication or studies of included studies[b]
**EUCTR2010-018412-32-ES**	Secondary publication or studies of included studies[b]
**EUCTR2010-018412-32-DE**	Secondary publication or studies of included studies[b]
**EUCTR2010-018412-32-BE**	Secondary publication or studies of included studies[b]
**NCT01396239**	Secondary publication or studies of included studies[c]
**EUCTR2010-024566-22-Outside-EU/EEA**	Secondary publication or studies of included studies[a]
**EUCTR2010-020069-26-PL**	Secondary publication or studies of included studies[e]
**EUCTR2010-020069-26-Outside-EU/EEA**	Secondary publication or studies of included studies[e]
**EUCTR2010-020069-26-NL**	Secondary publication or studies of included studies[e]
**EUCTR2010-020069-26-IT**	Secondary publication or studies of included studies[e]
**EUCTR2010-020069-26-HU**	Secondary publication or studies of included studies[e]
**EUCTR2010-020069-26-FR**	Secondary publication or studies of included studies[e]
**EUCTR2010-020069-26-ES**	Secondary publication or studies of included studies[e]
**EUCTR2010-020069-26-DK**	Secondary publication or studies of included studies[e]
**EUCTR2010-020069-26-DE**	Secondary publication or studies of included studies[e]
**EUCTR2010-020069-26-CZ**	Secondary publication or studies of included studies[e]
**EUCTR2010-020069-26-BE**	Secondary publication or studies of included studies[e]
**NCT01462292**	Secondary publication or studies of included studies[d]
**NCT01254019**	Secondary publication or studies of included studies[e]
**EUCTR2010-020069-26-NO**	Secondary publication or studies of included studies[e]
**EUCTR2014-002008-25-FR**	Secondary publication or studies of included studies[f]
**EUCTR2016-005000-26-Outside-EU/EEA**	Secondary publication or studies of included studies[c]
**EUCTR2010-024566-22-FR**	Secondary publication or studies of included studies[a]
**Goemans 2017[6]**	Secondary publication or studies of included studies[e]
**PMID:26086759**	Secondary publication or studies of included studies[f]
**NCT02310906**	Clinical trial registry with no result data
**EUCTR2014-005296-81-BE**	Clinical trial registry with no result data
**NCT02500381**	Clinical trial registry with no result data
**NCT02740972**	Clinical trial registry with no result data
**EUCTR2015-002069-52-BE**	Clinical trial registry with no result data
**EUCTR2015-002069-52-CZ**	Clinical trial registry with no result data
**EUCTR2015-002069-52-DE**	Clinical trial registry with no result data
**EUCTR2015-002069-52-ES**	Clinical trial registry with no result data
**EUCTR2015-002069-52-FR**	Clinical trial registry with no result data
**EUCTR2015-002069-52-GB**	Clinical trial registry with no result data

Secondary publications of: a, Flanigan 2014; b, Voit 2014; c, Mendell 2013; d, NCT01462292; e, NCT01254019; f, Muntoni 2017

1. Nakamura A, Takeda Si. Exon-skipping therapy for Duchenne muscular dystrophy. Lancet. 2011;378:546-7.
2. Goemans NM, Tulinius M, Van Den Akker JT, et al. Systemic administration of PRO051 in Duchenne's muscular dystrophy. N Engl J Med. 2011;364:1513-22.
3. Garralda ME, Kinali M, Cirak S, et al. Emotional impact of a paediatric exon-skipping therapy trial. Dev Med Child Neurol. 2011;53:1157-59.
4. Cirak S, Feng L, Anthony K, et al. Restoration of the dystrophin-associated glycoprotein complex after exon skipping therapy in duchenne muscular dystrophy. Mol Ther. 2012;20:462-67.
5. Rodino-Klapac LR. Microrna based treatment of cardiomyopathy: Not all dystrophies are created equal. J Am Heart Assoc. 2013;2.
6. Goemans N, Mercuri E, Belousova E, et al. A randomized placebo-controlled phase 3 trial of an antisense oligonucleotide, drisapersen, in Duchenne muscular dystrophy. Neuromuscul Disord. 2017; doi: 10.1016/j.nmd.2017.10.004.
